# Activation of the plant mevalonate pathway by extracellular ATP

**DOI:** 10.1038/s41467-022-28150-w

**Published:** 2022-01-21

**Authors:** Sung-Hwan Cho, Katalin Tóth, Daewon Kim, Phuc H. Vo, Chung-Ho Lin, Pubudu P. Handakumbura, Albert Rivas Ubach, Sterling Evans, Ljiljana Paša-Tolić, Gary Stacey

**Affiliations:** 1 Divisions of Plant Science and Biochemistry, Christopher S. Bond Life Sciences Center, University of Missouri, Columbia, MO 65211 USA; 2 Department of Forestry, University of Missouri, Columbia, MO 65211 USA; 3 Environmental Molecular Sciences Laboratory, Earth and Biological Sciences Directorate, Pacific Northwest National Laboratory, 902 Battelle Boulevard, Richland, WA 99354 USA; 4 Present Address: Inari Agriculture, NV, Industriepark Zwijnaarde 7A, 9052 Ghent, Belgium; 5 Present Address: School of Biomedical Engineering, International University, Vietnam National University, Ho Chi Minh City, 71300 Vietnam

**Keywords:** Plant immunity, Plant molecular biology, Plant signalling, Wounding, Secondary metabolism

## Abstract

The mevalonate pathway plays a critical role in multiple cellular processes in both animals and plants. In plants, the products of this pathway impact growth and development, as well as the response to environmental stress. A forward genetic screen of *Arabidopsis thaliana* using Ca^2+^-imaging identified mevalonate kinase (MVK) as a critical component of plant purinergic signaling. MVK interacts directly with the plant extracellular ATP (eATP) receptor P2K1 and is phosphorylated by P2K1 in response to eATP. Mutation of P2K1-mediated phosphorylation sites in MVK eliminates the ATP-induced cytoplasmic calcium response, MVK enzymatic activity, and suppresses pathogen defense. The data demonstrate that the plasma membrane associated P2K1 directly impacts plant cellular metabolism by phosphorylation of MVK, a key enzyme in the mevalonate pathway. The results underline the importance of purinergic signaling in plants and the ability of eATP to influence the activity of a key metabolite pathway with global effects on plant metabolism.

## Abstract (web summary)

Products of the mevalonate pathway support plant development. Here the authors show that the extracellular ATP receptor P2K1 phosphorylates mevalonate kinase and this affects the mevalonate pathway.

## Introduction

All living organisms produce isoprenoids and their diverse derivatives, which are essential for various cellular functions, through the mevalonate (MVA) pathway. In mammals, proper inhibition of 3-hydroxyl-3-methylglutaryl co-enzyme A reductase (HMGR) early in the mevalonate pathway is prescribed as a key treatment for hypercholesterolemia and cardiovascular disease^1–3^. Loss-of-function mutations in mevalonate kinase (MVK) cause two types of inflammatory disease, hyper-immunoglobulin D and periodic fever syndrome (HIDS) and mevalonate aciduria (MA)^4,5^. Specific inhibition of MVK increases oncogenic mutant p53 degradation, promoting suppression of cancer progression^6^. Fluvastatin, a HMGR inhibitor, suppresses the activity of the purinergic receptor P2X4 in monocytes through cholesterol depletion^7^. However, there is no evidence in animals for direct action of purinergic signaling on the MVA pathway. Besides the key role in cholesterol biosynthesis, the MVA pathway provides a wide range of compounds that impact diverse physiological functions, including membrane biogenesis, cell growth, and inflammation^2,3,8^.

In contrast to animals, less is known about the regulation of the MVA pathway in plants, although it clearly contributes to a wide range of cellular processes. As sessile organisms, plants must be able to grow in place and adapt to a variety of stresses brought about by pests, pathogens or changing environmental conditions. A large number of isoprenoids in the MVA pathway play important roles as mediators of interactions between plants and their environment, such as defense responses against biotic and abiotic stresses. Isopentenyl diphosphate (IPP), a universal precursor of isoprenoids, is used for biosynthesis of phytosterols and dolichols (e.g., N-glycosylation for membrane anchoring) that are essential components of the cell membrane and also the biosynthesis of isoprenoid-derived phytohormones (e.g., brassinosteroids and cytokinins), which regulate plant growth and development^9–11^. Farnesyl diphosphate (FPP) and geranylgeranyl diphosphate (GGPP) are used for protein prenylation that regulates various processes, such as meristem development, abscisic acid signaling, cytokinin biosynthesis, and innate immunity^12^. Isoprenes and terpenes, as volatile isoprenoids, are synthesized in plants for protection against pathogens and herbivores, or to communicate with their environment^13^. In addition, other isoprenoids, such as phytosterols and artemisinin, have medical value, including anti-cancer and anti-malaria activities^14,15^. Hence, the MVA pathway is central to a plethora of cellular processes and, therefore, its regulation is of critical importance.

A few *Arabidopsis* mutants have been reported with defects in the MVA pathway (see below). The initial reaction is catalyzed by *Acetoacetyl-CoA thiolase* (*AACT*). *Arabidopsis aact2* null mutants are embryo lethal, and RNAi silencing of *aact2* was found to reduce growth and apical dominance as well as altering the accumulation of sterols^16^. *Arabidopsis aact1* mutants have no visible phenotypic or metabolic changes. Interestingly AACT1 is localized in peroxisomes, indicating a possibly different metabolic role of AACT1 relative to AACT2^16^. The next enzyme in the pathway is 3-hydroxy-3-methylglutaryl-CoA synthase (HMGS). Knockdown mutation of *HMGS* results in deficient formation of the pollen coat, and *HMGS* knockout causes gametophyte lethality^17^. Two *3-hydroxy-3-methylglutaryl-CoA reductase* (*HMGR*) genes exist in the *Arabidopsis* genome. *hmgr1* mutant plants are male sterile and dwarf, and exhibit early senescence with sterol levels ~50% of wild-type^18,19^. While *hmgr2* mutants show no distinctive phenotypic differences compared to wild-type, *HMGR2* can partially complement the lost function of HMGR1 in *hmgr1* mutant^19^. Inhibition of HMGR reduces root growth and the production of key pathway products, such as sterols^20,21^. No visible phenotypes were observed in single mutants of either *Diphospho-mva Decarboxylase* (*MPDC*) *1* or *2*, whereas *mpdc1/2* double mutant plants exhibit ~20% unpollinated ovules in the siliques^22^. Mutants lacking isopentenyl diphosphate isomerase (IPPI) 1 and 2 exhibit dwarfism and male sterility, as well as reduced sterol and ubiquinone levels^23^. Loss of farnesyl diphosphate synthase (FPS) activity in *fps1* and *fps2* mutant plants blocks embryogenesis at the early globular stage and reduces sterol and ubiquinone levels^24^. Lack of geranylgeranyl diphosphate synthase (GGPPS) activity prevents embryo development^25^. Surprising given the level of research attention given to the MVA pathway, there are no reports of mevalonate kinase (*mvk*) mutants. Hence, it is unclear what phenotypes would be associated with the loss of this key, early enzyme in the pathway.

Cell surface-localized pattern recognition receptors (PRRs) recognize characteristic microbe or pathogen-associated molecular patterns (MAMPs or PAMPs) and host-derived damage associated patterns (DAMPs), activating pattern-triggered immunity to invading pathogens or pests^26–28^. Extracellular ATP (eATP) is recognized as a DAMP in both plants and animals where it can, for example, be released through cellular damage^26,29^. The first eATP receptor in plants, P2K1 (also termed DORN1 or LecRK-I.9) binds to ATP and ADP, leading to a variety of cellular changes, among them an increase in cytoplasmic Ca^2+^ levels, production of reactive oxygen species (ROS), and defense-related transcriptional responses^30–33^. Recent studies have begun to unravel the complex signaling events that mediate purinergic signaling in plants but progress is still at an early stage^34^. In order to further elucidate the details of the plant eATP signaling pathway, we performed a forward genetic screen based on the aequorin-bioluminescence Ca^2+^ imaging approach^35^. This resulted in the identification of an *Arabidopsis* mutant that was defective in Ca^2+^ influx triggered upon eATP addition. Subsequent positional cloning and complementation showed that this mutant phenotype was the result of the loss of mevalonate kinase (MVK) function. Subsequent research showed that MVK directly interacts with the eATP receptor P2K1 resulting in transphosphorylation of MVK and subsequent activation of the mevalonate pathway. These results further underline the ability of purinergic signaling to impact a wide variety of plant processes, similar to its impact on animal physiology.

## Results

### Cytosolic Ca^2+^ response in *24-14* mutant plants upon extracellular ATP treatment

**Fig. 1:**
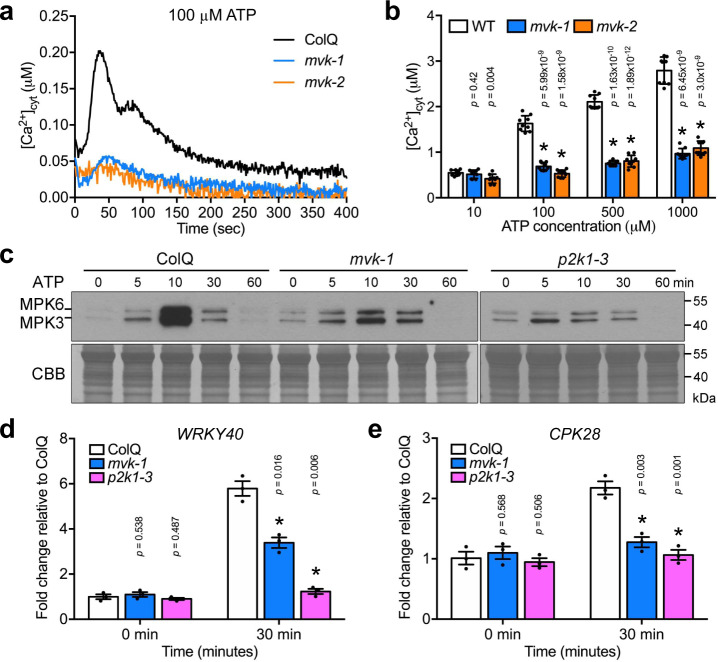
*mvk-1* mutants show lower response to extracellular ATP. **a** The kinetics of the cytoplasmic calcium response to 100 μM ATP for 400 sec in ColQ, *mvk-1*, and *mvk-2* (*mvk-2-4* line) plants. **b** The bar graph shows the integrated calcium response to 10, 100, 500, and 1000 μM of ATP for 400 sec in ColQ, *mvk-1*, and *mvk-2* (*mvk-2-4* line) mutant plants. Asterisks indicate significant differences between ColQ and *mvk* mutants plants (means ± SEM, *n* = 9 seedlings, *P < 0.001, two-sided Student’s *t* test). **c** The *mvk-1* mutant plant exhibits reduced phosphorylation of MPK3 and MPK6 in response to 100 μM of ATP compared to wild-type over a time-course from 0 to 60 min. Phosphorylation of MPK3 and MPK6 was detected using antibody against phospho-p44/p42 mitogen-activated protein kinase. *p2k1-3* mutant plants were used as a negative control. The coomassie brilliant blue (CBB) staining (bottom panel) showed equal loading. **d**, **e** Relative expression of *WKRY40* and *CPK28* in 10-day-old ColQ, *mvk-1*, and *p2k1-3* whole seedlings treated with 100 μM ATP for 30 min was performed using qRT-PCR analysis. Gene expression data were normalized using the *SAND* reference gene. Bar graphs represent means of three pooled biological replicates. Asterisks indicate the significant differences compared to ColQ at the same time points (*P < 0.05, two-sided Student’s *t* test). All above experiments were repeated three times with similar results.

The same genetic screen that ultimately led to the identification of P2K1 as an eATP receptor identified a number of additional mutations that affected the eATP-induced cytoplasmic calcium response, but did not map to the *p2k1* gene^30^. This assay utilized transgenic *Arabidopsis* plants (hereafter ColQ) expressing the calcium reporter protein aequorin^36^. Among the mutants that showed a reduced calcium response to eATP addition was line *24-14* (referred to as *mvk-1*) (Fig. 1a, b). Unlike wild-type and *p2k1* mutant plants, mutant line *24-14* plants exhibited short roots and small leaves (Supplementary Fig. 1a).

We previously showed that eATP elicits the activation of mitogen-activated protein kinases (MAPKs)^30,37^. In order to confirm whether the *24-14* mutant also failed to trigger eATP induced MAPKs signaling, we checked mitogen-activated protein kinase 3 and 6 (MPK3/6) phosphorylation. Addition of ATP to *24-14* mutant plants resulted in a significant decrease in the phosphorylation of MPK3/6 in a manner similar to that found with the eATP receptor mutant *p2k1-3*, while strong activation of MPK3/6 was seen in wild-type plants (Fig. 1c).

The addition of eATP also triggers a strong transcriptional response^30,31^. Two genes that respond to ATP are *wrky domain transcription factor 40* (*WRKY40*) and calcium-dependent protein kinase *28* (*CPK28*). While both of these genes were strongly up-regulated in the wild-type upon eATP elicitation, their expression was only slightly up-regulated in the *24-14* mutant (Fig. 1d, e). Thus, with regard to the cellular calcium response, MAPK activation and gene expression, the *24-14* mutant line showed clear disruption of normal purinergic signaling.

### A mutation in the *MVK* gene is responsible for the phenotype of the *24-14* mutant line

In order to identify the gene responsible for the reduction in purinergic signaling in the *24-14* mutant (subsequently referred to as *mvk-1*), we performed map-based cloning and whole-genome Illumina sequencing. These results mapped the *mvk-1* mutation to a 146-kb interval on the short arm of chromosome 5 (Supplementary Fig. 1b, c). Subsequent whole-genome sequencing identified a substitution mutation (G133A; Ala45Ser) in the gene, *At5g27450*, predicted to encode mevalonate kinase (MVK) (Supplementary Fig. 1c–e). This mutation is located at the end of the first exon of *mvk* gene resulting in failure to splice the first intron, as well as a deletion of 28 base-pairs in the *MVK* transcript. In the *mvk-1* mutant, two *mvk* transcripts were observed, whereas no wild-type *MVK* transcript was detected (Supplementary Fig. 2a). These defects lead to premature stop codons (Supplementary Fig. 2b). To confirm the *MVK* mutation was responsible for the *mvk-1* phenotype, we next complemented the mutant phenotype. Expression of the wild-type *MVK* driven either by the *MVK* native promoter or *CaMV 35* *S* promoter rescued both the *mvk-1* mutant calcium influx in response to ATP and reduced growth phenotypes (Supplementary Fig. 3a–d, g).

In order to characterize the expression patterns of *MVK*, transgenic *Arabidopsis* plants were constructed where the *MVK* gene fused to the β-glucoronidase (*GUS*) reporter gene was expressed from the native promoter. GUS activity was ubiquitously detected, especially in highly proliferative tissues, including root and shoot apical meristems, as well as in young seedling leaves (Supplementary Fig. 4a–f), similar to a previous report^38^.

A search of the literature and related databases failed to identify any available *mvk* mutants in *Arabidopsis*^22^. Therefore, in order to verify the observed phenotype of the *mvk-1* mutant, we disrupted the *MVK* gene using CRISPR/Cas9 technology (Supplementary Fig. 5a, b). Consistent with the phenotype of the *mvk-1* mutant line, *mvk*-CRISPR/Cas9 (hereafter *mvk-2*) mutant plants did not show an increase in intracellular calcium concentration upon eATP treatment (Fig. 1a, b; Supplementary Fig. 5c).

### *Arabidopsis* MVK enzymatic activity

**Fig. 2:**
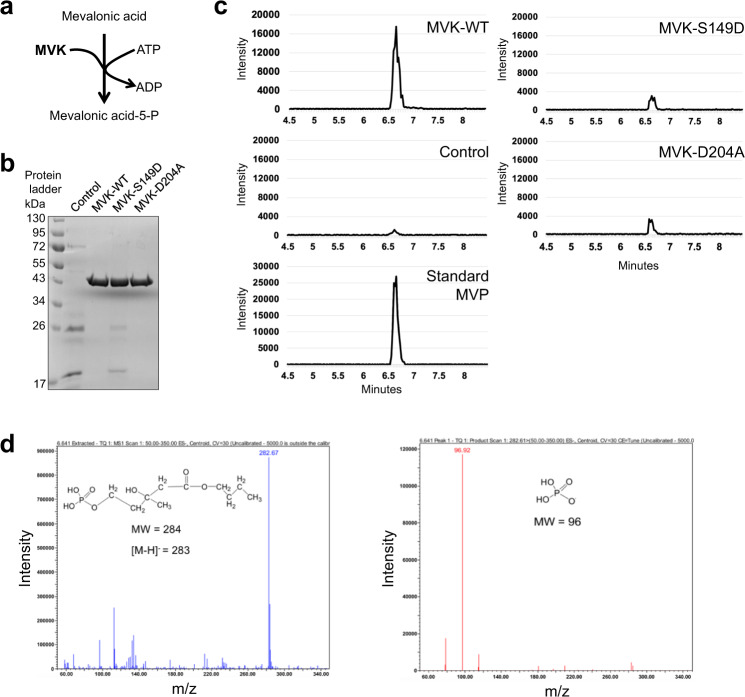
Enzymatic activities of recombinant mevalonate kinase (MVK), and identification of reaction products. **a** Schematic representation of the conversion of mevalonic acid (MVA) into mevalonic acid-5-phosphate (MVP) (consuming ATP). **b** SDS-PAGE gel showing His-tag purified proteins expressed in *E*. *coli* cells expressing His-tagged MVK-WT, and two dead-versions of MVK (MVK-S149D, and MVK-D204A). As control, proteins were purified from cells carrying empty vector. The protein was measured by Coomassie brilliant blue staining. Experiment was repeated three times with similar results. **c** The ion chromatogram of MVP butyl ester in the samples (MVK-WT, control, MVK-S149D, MVK-D204A, and Standard MVP) quantified by LC-MS/MS analysis (m/z 283→96). MVP+ MVK-WT peak (~6.6 min) is detected in MVK reaction but not in control (empty vector control cells), and dead-versions of MVK. **d** The ion spectra of the molecular ion (m/z 283) (left) and product ions (right) for the analysis of the MVP butyl ester ionized in negative ion mode (ES^−^).

Previous studies in yeast, fungi, plants, and mammals demonstrated that MVK enzymatically coverts mevalonic acid (MVA) to mevalonic acid-5-phosphate (MVP) at an early step in the MVA pathway (Fig. 2a; Supplementary Fig. 1e)^39,40^. Based on the information of critical conserved motifs in other MVKs from previous studies in human and mosquito, we generated mutated versions of MVK (S149D, D204A) as negative controls^41,42^. In order to ascertain whether MVK (*At5g27450*) has enzymatic activity, we recombinantly expressed and affinity purified wild-type and mutated versions of MVK proteins from *E. coli*. Recombinant MVK, mutated versions of MVK, and control proteins were reacted with MVA and ATP, then their enzymatic activity was analyzed using ultra-performance liquid chromatography (UPLC) (Fig. 2b–d; Supplementary Fig. 6). The enzymatic products of wild-type MVK showed the presence of MVP (~6.6 mins), identical to standard MVP (~6.6 mins), whereas no MVP was detected in empty vector control samples, while the MVK-S149D and D204A MVK proteins showed very weak MVP production (Fig. 2c). These results indicate that *Arabidopsis* MVK has enzymatic activity.

### *mvk-1* mutant plants show a strong alteration in the level of metabolites from the MVA pathway

**Fig. 3:**
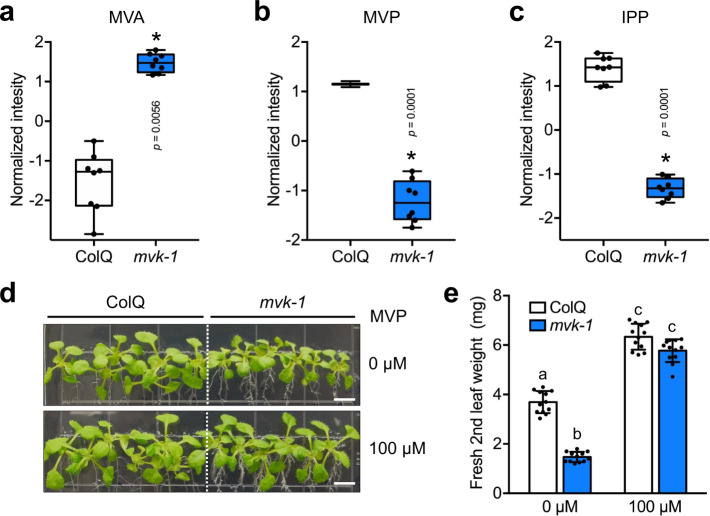
*mvk-1* mutant plants show reduced mevalonic acid-5-phosphate (MVP) levels. Exogenous application of MVP partially restores the altered *mvk-1* mutant phenotype. **a**–**c** Box-and-whisker plots of intermediate compounds in MVA pathway showing their relative abundances. ColQ and *mvk-1* mutant root extracts were analyzed with a high-resolution mass spectrometer (HRMS) Orbitrap Velos (Thermo Fisher Scientific) coupled to a Thermo Vanquish HPLC (High Pressure Liquid Chromatographer; Thermo Fisher Scientific). MVA, Mevalonic acid; MVP, Mevalonic acid-5-phosphate; IPP, isopentenyl diphosphate. Asterisks indicate significant differences between ColQ and *mvk-1* (means ± SEM, *n* = 8 biological replicates, *P < 0.05, two-sided Student’s *t* test). Box-and-whisker plots show max and min, 25–75th percentiles (box), and median (center line). Experiment was repeated three times with similar results. **d** 16-day-old ColQ and *mvk-1* mutant seedlings grown on medium without or with 100 µM mevalonic acid-5-phosphate (MVP). Scale bars: 0.5 cm. **e** Fresh second leaf weight of 16-day-old plants (*n* = 21 leaves for ColQ and *n* = 24 leaves for *mvk-1*). Data represent mean ± SEM from independent experiments. Means with different letters are significantly different (*P* < 0.05). *P* values indicate significance relative to ColQ with mock treatment and were determined by one-sided ANOVA with multiple comparisons and adjusted using a Duncan post hoc test. Experiment was repeated three times with similar results.

Loss of MVK activity in mammals leads to accumulation of MVA and deficiency in downstream compounds for which MVA serves as a precursor^43^. Due to the changes in downstream compounds in the MVA pathway, *Arabidopsis* mutants in this pathway show various physiological phenotypes, such as dwarfism, gametophytic male sterility, small leaves, shorter hypocotyl and roots^16,17,19,23^. It was also shown that application of the HMGR inhibitor, lovastatin, inhibits plant growth^20^. Therefore, one would predict that mutation of *MVK* should strongly alter the levels of downstream MVA pathway metabolites. In order to confirm this prediction, we performed comparative metabolomic analysis of wild-type and *mvk-1* mutant roots harvested from 14-day-old seedlings. As expected, the *mvk-1* mutant plants showed a marked reduction in MVP levels (Fig. 3b), while MVA, a precursor compound of MVP, was significantly higher in the mutant (Fig. 3a). The levels of key downstream metabolites, such as Isopentenyl diphosphate (IPP), were also significantly reduced in the *mvk-1* mutant plants (Fig. 3c). A full list of the metabolites detected in our analysis is shown in Supplementary Data 1.

If the lack of MVP was solely responsible for phenotypes seen in the *mvk-1* mutants (Fig. 1; Supplementary Fig. 1a), then it might be possible to reverse these phenotypes by simply feeding the plants with MVP. Therefore, we first tested the growth response of the *mvk-1* mutants on MS medium supplemented with 100 µM MVP. The growth of *mvk-1* and *mvk-2* mutant plants was partially rescued by exogenous application of MVP, demonstrated by seedling growth and also leaf fresh weight (Fig. 3d, e; Supplementary Fig. 5d, e). We hypothesized that if output compounds of the MVA pathway are associated with eATP signaling, application or blocking of the MVA pathway would cause alterations in the ATP-induced calcium response. Indeed, *mvk-1* seedlings, pre-treated with MVP for 1 h and subsequently treated with ATP, were partially rescued with regard to the eATP-induced cytoplasmic calcium response (Supplementary Fig. 7a, b).

As mentioned previously, lovastatin is an inhibitor of HMG-CoA reductase (HMGR)^1,20^. Therefore, we treated seedlings for 1 h with lovastatin and subsequently challenged with eATP. The application of lovastatin strongly reduced the eATP-induced cytoplasmic calcium response in wild-type but not in *mvk-1* plants (Supplementary Fig. 7c, d). Together, these results suggest that metabolites downstream of MVA modulate the cellular calcium response to eATP and that this effect, and not some indirect response, is what determines the observed phenotype of the *mvk-1* mutant plants.

### MVK is involved in the ATP signaling pathway

**Fig. 4:**
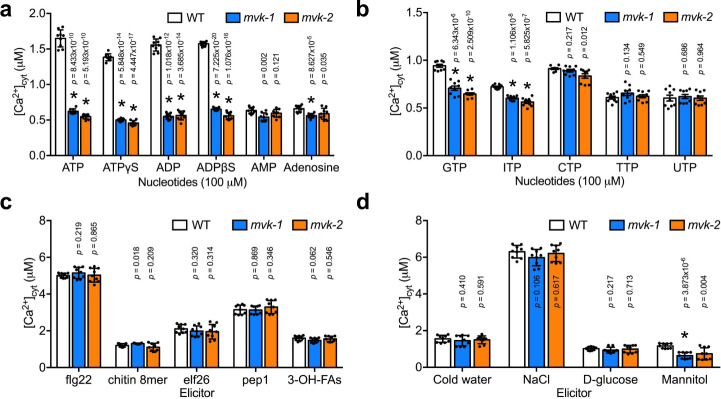
The *mvk* mutants show defects in the calcium response to various nucleotides. **a** Calcium responses to adenine nucleotides and non-hydrolysable derivatives in ColQ, *mvk-1*, and *mvk-2* (*mvk-2-4* line) plants. **b** Calcium responses to other nucleotides in ColQ, *mvk-1*, and *mvk-2* (*mvk-2-4* line) plants. **c**, **d** Biotic (100 nM of flg22, chitin, elf26, and pep1; 5 µM 3-OH-FAs) and abiotic stress reagents (ice-chilled water, 300 mM NaCl, 5% D-glucose, and 300 mM mannitol) induced calcium responses in *mvk* mutants. Asterisks indicate significant differences between ColQ and *mvk* mutants plants. All data represented as means ± SEM, *n* = 9 seedlings (*P < 0.001, two-sided Student *t* test). All above experiments were repeated three times with similar results.

The plant eATP receptor P2K1 displays higher preference for purine nucleotides than pyrimidine nucleotides^30^. Therefore, to see whether *mvk-1* mutants show a specific response to purine nucleotides, we checked the calcium response of *mvk-1* mutant plants to a variety of nucleotides. Both *mvk-1* and *mvk-2* mutant plants exhibited defects in the cytoplasmic calcium response to purine nucleotides, such as poorly hydrolyzed ATP analogs (ATPγS and ADPβS), ADP, GTP, and ITP, whereas the *mvk-1* and *mvk-2* mutants showed no difference in response to pyrimidine nucleotides, CTP, TTP, and UTP compared with wild-type plants (Fig. 4a, b). We also tested the nucleotide induced calcium responses in *Arabidopsis* plants overexpressing *MVK* driven by the strong CaMV 35 S promoter (*p35S::MVK*) in the wild-type background. The *p35S::MVK* lines showed ~30% higher calcium influx than wild-type plants in response to ATP (Supplementary Fig. 3e, f). The intracellular calcium responses of the *mvk-1* mutants to various biotic (flg22, chitin, elf26, pep1, and 3-OH-FAs) and abiotic (cold water, NaCl, D-glucose, and mannitol) calcium elicitors were similar to those of wild-type plants (Fig. 4c, d). Consistent with these results, *mvk-2* mutant plants also responded similar to the wild type to these the same elicitors (Fig. 4c, d). Overall, our results suggest that *MVK* plays an important role in the response to purine nucleotides, likely mediated through the action of the P2K1 receptor.

As previously shown, eATP treatment and wounding induce overlapping transcriptional responses^30^. Therefore, we tested whether both eATP and wounding induce expression of *MVK*. Indeed, we found that *MVK* expression was induced by eATP over a time-course and also by wounding of plants, as measured by *pMVK::GUS* expression (Supplementary Fig. 4g, h).

### MVK interacts with P2K1 *in planta*

**Fig. 5:**
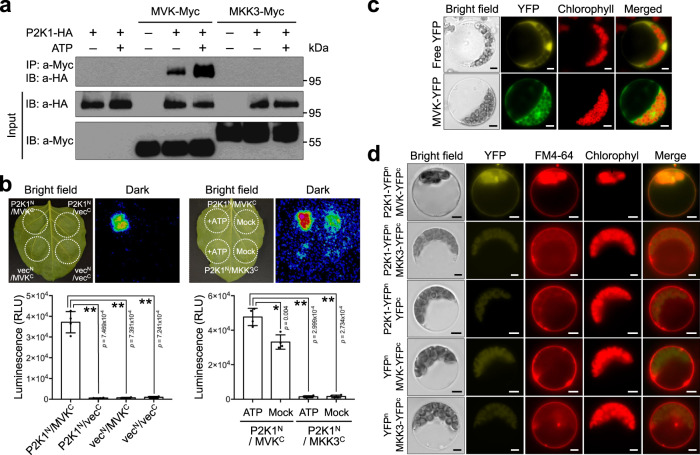
MVK interacts with P2K1 in vivo. **a** Co-immunoprecipitation of P2K1 and MVK proteins. The indicated constructs were transiently co-expressed in *Nicotiana benthamiana* leaves infiltrated with either 200 μM ATP for 30 min (+) or 2 mM MES (pH 5.7) as a control (−). Full-length MKK3 was used as a negative control. Co-IP was performed using anti-HA and anti-Myc antibodies. **b** Split-luciferase assay image of *N. benthamiana* leaves co-infiltrated with *Agrobacterium* strains containing P2K1^N^/MVK^C^, P2K1^N^/vec^C^, vec^N^/MVK^C^, vec^N^/vec^C^, and P2K1^N^/MKK3^C^ (Negative control). Dotted circles indicate leaf panels that were infiltrated with *Agrobacterium* carrying the respective constructs. +ATP, Leaves infiltrated with 200 µM ATP; Mock, Leaves infiltrated with 2 mM MES (pH 5.7). Quantification of relative luminescence unit (RLU) intensity of P2K1^N^/MVK^C^. Asterisks indicate significantly different from P2K1^N^/vec^C^, vec^N^/MVK^C^, and vec^N^/vec^C^ (left bottom, *n* = 4 leaves; **P < 0.01, two-sided Student’s *t* test) or negative control P2K1^N^/MKK3^C^ (right bottom, *n* = 4 leaves; *P < 0.01, **P < 0.001, two-sided Student’s *t* test). **c** Subcellular localization of MVK in *Arabidopsis* protoplast. *Arabidopsis* MVK fused to YFP shows cytosolic localization. Free YFP was used as a control. Scale bars: 5 µm. **d** Biomolecular fluorescence complementation (BiFC) assay in Arabidopsis protoplasts. The indicated constructs were transiently expressed in wild-type protoplasts and the BiFC assay was performed. See also the quantification of relative fluorescence intensity shown in Supplementary Fig. 8. FM4-64 was applied to stain the plant plasma membrane. MKK3 was used as a negative control. Scale bars: 5 µm. All above experiments were performed and analyzed three times with similar results.

Unlike the eATP receptor P2K1, which localizes to the plasma membrane^44^, MVK is predicted to localize in the cytosol (SUBA; http://suba.plantenergy.uwa.edu.au)^22^. Indeed, a previous study showed that *Arabidopsis* MVK localized to the cytosol when expressed in *Catharanthus roseus* cells^45^. In order to confirm MVK localization in *Arabidopsis* cells, we expressed a YFP fluorophore tagged version of MVK in *Arabidopsis* protoplasts, MVK was confined to the cytosol (Fig. 5c). Therefore, if MVK plays a role in eATP signaling pathway, it could be via direct interaction with the eATP receptor. In order to test this hypothesis, we co-expressed HA-tagged P2K1 in *Nicotiana benthamiana* leaves with a Myc-tagged MVK with or without the addition of ATP (Fig. 5a). Subsequent co-immunoprecipitation (Co-IP) showed a clear interaction of MVK and P2K1 that was enhanced by eATP. Similarly, use of the split-luciferase complementation imaging (LCI) assay in *N. benthamiana* leaves also showed interaction between MVK and P2K1, which was enhanced by eATP (Fig. 5b). The biomolecular fluorescence complementation (BiFC) assay in *Arabidopsis* protoplasts also showed that MVK specifically interacts with P2K1 on the plasma membrane (Fig. 5d; Supplementary Fig. 8). The cytoplasmic kinase, mitogen-activated protein kinase kinase 3 (MKK3)^46^, was used as a negative control in these assays and showed no interaction with P2K1 (Fig. 5). Together, the results suggest that MVK directly interacts with the P2K1 receptor on the plasma membrane and that this interaction is strengthen by the addition of eATP.

### MVK is phosphorylated by P2K1

**Fig. 6:**
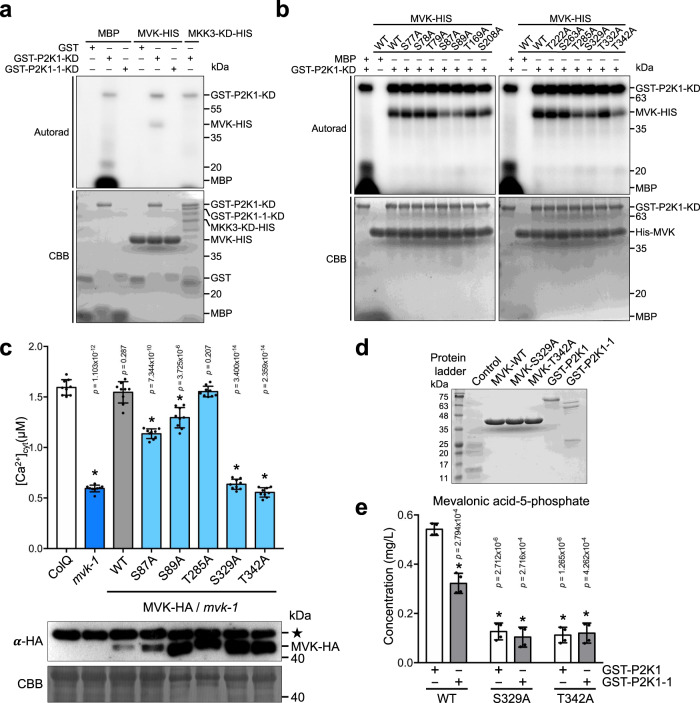
P2K1 phosphorylates MVK in vitro. MVK residues S329 and T342 are required for ATP-triggered calcium production. **a** P2K1 phosphorylates MVK. Purified MVK-HIS recombinant protein was incubated with GST-P2K1-KD kinase domain, GST-P2K1-1-KD (kinase dead), or GST in an in vitro kinase assay. Autophosphorylation and trans-phosphorylation were measured by incorporation of γ-[32 P]-ATP. MBP and MKK3-KD-HIS kinase domain were used as positive and negative controls, respectively. Protein loading was visualized by Coomassie brilliant blue (CBB) staining. **b** P2K1 phosphorylates MVK and site-directed mutation of P2K1-mediated MVK phosphor sites. Purified MVK-HIS recombinant protein was incubated with GST-P2K1-KD kinase domain in an in vitro kinase assay. Autophosphorylation and trans-phosphorylation were measured by incorporation of γ-[32 P]-ATP. MBP was used as positive control. Protein loading was visualized by Coomassie brilliant blue (CBB) staining. **c** MVK phosphor sites are required for ATP-triggered calcium production. The indicated constructs were expressed in the *mvk-1* mutant background and treated with 100 μM ATP. All data represented as means ± SEM, *n* = 9 seedlings (*P < 0.05, two-sided Student’s *t* test). See also the calcium kinetics shown in Supplementary Fig. 9b. Total MVK-HA protein was detected by anti-HA immunoblot. Star indicates nonspecific band which was used as a loading control. **d** Recombinant MVK and P2K1 proteins. SDS-PAGE of HIS and GST tagged proteins isolated from *E. coli* cells expressing recombinant control (empty vector), MVK-WT-HIS, MVK-S329A-HIS, MVK-T342A-HIS, GST-P2K1-KD, and GST-P2K1-1-KD. Protein loading was visualized by coomassie brilliant blue staining. **e** Enzymatic activity of P2K1 and MVK-WT, MVK-S329A, and MVK-T342A proteins measured by UPLC-MS/MS. Data are shown as mean ± SEM. Asterisks indicate significant differences between WT and MVK proteins (S329A and T342A) with GST-P2K1 or GST-P2K1-1. (*n* = 4 biological replicates, *P < 0.01, two-sided Student’s *t* test). All above experiments were performed and analyzed three times with similar results.

It was previously demonstrated that the kinase activity of P2K1 is essential for purinergic signaling *in planta*^30,33^. Therefore, we tested whether P2K1 can phosphorylate MVK. Full-length MVK protein, the P2K1 kinase domain (P2K1-KD) or a P2K1 kinase-dead version (P2K1-1-KD) were recombinantly expressed and purified. Recombinant MVK protein was incubated with the purified P2K1-KD and P2K1-1-KD and assayed for phosphorylation by radiolabeling with γ-[32 P]-ATP. The results show that the purified P2K1 kinase domain (KD) strongly trans-phosphorylated MVK in vitro, whereas the kinase-dead version of GST-P2K1-1-KD failed to phosphorylate MVK (Fig. 6a).

Mass spectrometric analysis of in-solution-trypsin digested peptides identified 13 possible in vitro MVK sites phosphorylated by P2K1 (Supplementary Fig. 9a). MVK is composed of three domains, the Galacto, Homoserine, Mevalonate, and Phosphomevalonate (GHMP) kinase N- and C-terminal, and the ATP binding domain (Supplementary Fig. 9a). In the GHMP kinase N-terminal region of MVK, two sites targeted by P2K1 were identified (T169, S208), and three sites (S329, T332, T342) were identified in the GHMP kinase C-terminal region, while eight other sites (S77, S78, T79, S87, S89, T222, S263, T285) were identified outside of GHMP domains (Supplementary Fig. 9a). In order to test whether the identified phosphorylated sites in MVK are important for activity or regulation of this enzyme, the 13 phospho-residues were each substituted with alanine to eliminate the possibility of trans-phosphorylation. Subsequent assays showed that, of these 13 residues, proteins having the MVK-S87A, S89A, T285A, S329A, or T342A mutation showed reduced phosphorylation in the presence of P2K1 (Fig. 6b). In order to investigate the relevance of this phosphorylation to MVK-mediated cytoplasmic calcium influx in response to ATP, wild-type MVK, as well as the S87A, S89A, T285A, S329A, or T342A MVK proteins were each fused to the HA epitope tag and, subsequently ectopically expressed in *mvk-1* mutant plants. Plants expressing the phospho-null S87A or S89A MVK proteins showed partial complementation of the ATP-induced cytoplasmic response upon addition of eATP. Plants expressing either S329A or T342A MVK proteins showed no complementation (Fig. 6c; Supplementary Fig. 9b). Expression of the T285A MVK protein fully restored the wild-type phenotype (Fig. 6c; Supplementary Fig. 9b). From this analysis, we conclude that phosphorylation of S329 and/or T342 is critical for MVK function. Note that in every case, western blotting showed strong and roughly similar expression of the recombinant proteins (Fig. 6c).

In order to further confirm whether P2K1-mediated MVK phosphorylation affects MVK enzymatic activity, we combined the recombinant proteins in vitro and then measured MVK enzymatic activity (Fig. 6d, e). Production of MVP was significantly higher when MVK-WT-HIS was in the presence of GST-P2K1-KD, relative to similar experiments performed with the kinase-dead version GST-P2K1-1-KD (Fig. 6e). Production of MVP by MVK-S329A-HIS or MVK-S342A-HIS in the presence of either GST-P2K1-KD or GST-P2K1-1-KD was significantly lower relative to experiments performed with the wild-type MVK protein (Fig. 6e). Overall, these results suggest that MVK is a direct phosphorylation target of P2K1, and S329 and T342 are the critical MVK residues directly phosphorylated by P2K1 that modulate enzymatic activity.

### MVA pathway links with the ATP signaling pathway

Given that MVK directly interacts with and is phosphorylated by P2K1 in an ATP dependent manner, we investigated whether P2K1 accumulation is regulated by MVK. A previous study by Chen et al., 2017 showed that eATP could induce both P2K1 protein accumulation and autophosphorylation^33^. Thus, we expressed P2K1-HA in both wild-type and *mvk-1* mutant background plants. Western blotting showed that P2K1-HA protein levels in *mvk-1* plants were reduced, relative to wild type, at 0 min (Supplementary Fig. 10a). Interestingly, the levels of phosphorylated P2K1-HA were significantly decreased in *mvk-1* mutant plants at 15 and 60 min after ATP treatment (Supplementary Fig. 10a). Gel migration of the phosphorylated P2K1 protein was shifted by treatment with lambda protein phosphatase (lambda PP) to release any phosphate groups (Supplementary Fig. 10a).

In order to further understand whether the MVA pathway is involved in ATP signaling, we examined the ATP responses in the another MVA pathway mutant. *hmgr1-1* mutant plants lacking HMG-CoA reductase showed a reduced calcium response to eATP addition (Supplementary Fig. 10c, d). MPK3/6 phosphorylation and expression of ATP-induced genes were significantly reduced in *hmgr1-1* mutant (Supplementary Fig. 10e–g). In addition, treatment of *Arabidopsis* plants with lovastatin, an inhibitor of HMG-CoA reductase, decreased P2K1 accumulation and phosphorylation in wild-type and *mvk-1* mutant plants (Supplementary Fig. 10b). These results suggest that MVK regulates activation of P2K1 protein in an ATP-dependent manner and the MVA pathway, likely via downstream metabolite products, impacts the ATP signaling pathway.

### P2K1-mediated MVK phosphorylation plays an essential role in plant innate immunity

Activation of MAPK signaling is a critical aspect of the pathogen response pathway^47^. Previous studies reported that the P2K1 receptor is involved in plant defense against pathogens^33,48–52^. *Arabidopsis* responds to eATP with activation of MAPK phosphorylation^30^. In addition, application of eATP led to enhanced resistance to the bacterial pathogen *Pseudomonas syringae* via P2K1 signaling^33^. Given that the reduced activation of MPK3/6 in the *mvk-1* mutant background (Fig. 1c) and the interaction between MVK and P2K1 (Fig. 5), it seemed likely that MVK activation by P2K1 would also play a role in the plant innate immune response.

**Fig. 7:**
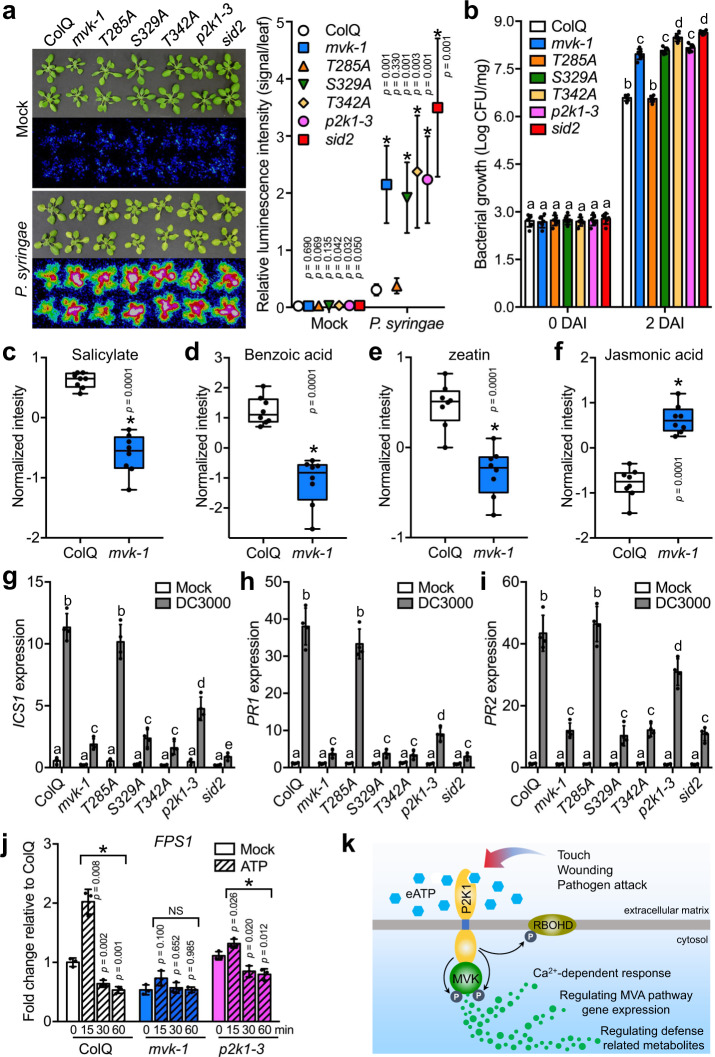
P2K1 mediated MVK phosphorylation at S329 and T342 plays a critical role in plant innate immunity. Altered *ICS1*, *PR1*, *PR2*, and *FPS1* gene expression and metabolites in *mvk-1* mutant plants. **a**, **b** ColQ, *p2k1-3* and *sid2* were used as controls in comparison to *mvk-1* mutant plants, as well as complemented *mvk-1* plants expressing the MVK-T285A, MVK-S329A, and MVK-T342A mutant protein. 3-week-old plants were flood inoculated with a *P. syringae* DC3000 lux suspension (OD_600_ = 0.002) containing 0.025% (v/v) Silwet L-77. **a** At two day after inoculation (DAI), bacteria invasion was detected by CCD camera (left panel). Quantification of relative luminescence intensity (signal/leaf) of infected leaf from *P. syringae* DC3000 lux (right panel). Values represent the mean ± SD. The asterisks indicate statistical significance (*n* = 6 seedlings, *P < 0.01, two-sided Student’s *t* test). **b** bacterial colonization was determined by plate counting (*n* = 12). Data represent mean ± SEM from independent experiments. Two-way ANOVA with Tukey’s multiple comparisons analysis were calculated by GraphPad Prism 7. Means with different letters are significantly different (*P* < 0.05). **c**–**f** Box-and-whisker plots of significant metabolites showing their relative abundances in ColQ and *mvk-1* plants. Root extracts were analyzed with a high-resolution mass spectrometer (HRMS) Orbitrap Velos coupled to a Thermo Vanquish HPLC. Asterisks indicate significant differences between ColQ and *mvk-1* (means ± SEM, *n* = 8 biological replicates, *P < 0.05, two-sided Student’s *t* test). Box-and-whisker plots show max and min, 25–75th percentiles (box), and median (center line). **g**–**i** *ICS1*, *PR1*, and *PR2* genes expression pattern in response to *P. syringae* DC3000. 3-week-old ColQ, *mvk-1*, *T285A*, *S329A*, *T342A*, *p2k1-3*, and *sid2* plants were treated with 2 mM MES (mock) and *P. syringae* DC3000 (OD_600_ = 0.002) for 24 h, then qRT-PCR analysis was performed. Expression of *ICS1, PR1*, and *PR2* was normalized using *SAND* reference gene. The results are relative to expression levels of mock treated plants. Two-way ANOVA analysis was calculated by GraphPad Prism 7. Means with different letters are significantly different (*n* = 4 biological replicates, *P* < 0.0001). **j** *FPS1* gene expression pattern in response to ATP. 10-day-old ColQ, *mvk-1*, and *p2k1-3* whole seedlings were treated with 2 mM MES (mock) and 100 μM ATP for 0, 15, 30, and 60 min, then qRT-PCR analysis was performed. Expression of *FPS1* was normalized using *SAND* reference gene. The results are relative to expression levels of ColQ mock treated plants (set as 1). Data represent mean ± SEM from independent experiments. The bar graphs are means of three biological repeats. Asterisks indicate the significant differences compared to each mock (*P < 0.05; NS, not significant; two-sided Student’s *t* test). **k** Model illustrating the proposed role of MVK in the extracellular ATP signaling pathway. When extracellular ATP bind to P2K1 receptor, MVK is phosphorylated and activated by P2K1 and in turn regulates Ca^2+^-dependent response, MVA pathway gene expression, and defense related metabolites. RBOHD is also phosphorylated by P2K1^33^. All above experiments were repeated three times with similar results.

To investigate the involvement of MVK in plant innate immunity, we challenged plants with the pathogen *P. syringae* DC3000 lux. *Arabidopsis salicylic acid induction-deficient 2* (*sid2*) and *p2k1-3* mutant plants were used as susceptible controls^34,53^. As predicted, these experiments showed that *mvk-1* mutant plants were more susceptible to the bacterial pathogen than wild-type plants (Fig. 7a). Consistent with the bio-luminescence assay, direct bacterial counts also confirmed higher pathogen numbers on *mvk-1* mutant plants relative to wild-type plants (Fig. 7b). In order to further understand whether the phosphor-null mutants S329A and T342A exhibit increased pathogen susceptibility, we also challenged these mutant plants with *P. syringae*. T285A was used as a complementation control. Both phosphor-null mutants were susceptible to the bacterial pathogen (Fig. 7a). In addition, bacterial growth was significantly increased in the both S329A and T342A mutant plants compared to wild-type (Fig. 7b). These results demonstrate that MVK is associated with P2K1 mediated innate immunity in *Arabidopsis*.

Salicylic acid (SA) is known for its role in initiating defense responses against pathogens such as *P. syringae*^54^. It was reported that reduced cytokinin, an isoprenoid-derived phytohormone, increased disease susceptibility to *P. syringae*, and cytokinin acts synergistically with SA to activate the expression of SA-related defense genes such as *isochorismate synthase* (*ICS1*) and *pathogenesis-related* (*PR*)^55^. Given that *mvk-1* plants showed reduced levels of IPP (Fig. 3c), it seemed possible that increased pathogen susceptibility in *mvk-1* plants is due to a reduction of MVA downstream metabolites, SA biosynthesis, or mis-regulation of defense-related genes expression. To better understand how MVK contributes to bacterial pathogen resistance, we performed metabolomic analysis of 2-week-old wild-type and *mvk-1* mutant plant roots. We found that SA was reduced in *mvk-1* mutant plants (Fig. 7c). Benzoic acid, a direct precursor of salicylic acid, was strongly reduced in *mvk-1* mutant plants (Fig. 7d). Zeatin, the most abundant cytokinin, was decreased in the *mvk-1* mutant (Fig. 7e). In contrast, JA levels were slightly higher in *mvk-1* mutant plants (Fig. 7f).

*ICS1* is one of the major enzymes involved in SA accumulation in response to *P. syringae* treatment^56^. Expression of the *PR* genes, a molecular marker associated with SA-regulated signaling response, is increased after *P. syringae* infection^53^. To examine the expression of *ICS1* and *PRs*, wild-type, *mvk-1*, and phosphor-null S329A and T342A mutants were treated with *P. syringae*. *p2k1-3* and *sid2* mutant plants were used as susceptible controls. T285A plants were used as a complementation control. The expression levels of *ICS1*, *PR1*, and *PR2* were similar in all mock-treated tissues 24 h after *P. syrinage* treatment (Fig. 7g–i). However, the levels of *ICS1*, *PR1*, and *PR2* were significantly lower in *mvk-1* and phosphor-null mutants than wild-type and T285A plants in response to *P. syringae* (Fig. 7g–i). Taken together, we conclude that, at least in part, the observed reduction in zeatin and SA levels, as well as downregulation of the defense-related genes, likely contributes to the increased susceptibility of *mvk-1* mutant plants to *P. syringae* DC3000.

Plants perceive a change in isoprenoids by down-regulation of *farnesyl diphosphate synthase* (*FPS*) in the MVA pathway (Supplementary Fig. 1e) as a stress signal, leading to misregulation of genes involved in abiotic and biotic stress responses, which affects the level of metabolites related to wounding and/or stress response [e.g., jasmonic acid (JA), and SA]^57^. Similar to wounding, eATP also induced the expression of *MVK* (Supplementary Fig. 4g, h). Previous transcriptome data showed that *FPS1* expression was increased by wounding at an early time point (at 15 min), then down-regulated over a time-course^58^. In addition, previous ATP treated transcriptome data revealed that eATP could affect the expression of *FPS1*^30^. Interestingly, these data showed that *FPS1* transcript levels in wild-type plants were down-regulated at 30 min post ATP treatment compared to mock treated plants. However, no statistical difference in the level of *FPS1* was observed in *p2k1* mutant plants compared to mock-treated wild-type plants^30^. To confirm these observations, we directly measured *FPS1* expression in wild-type, *mvk-1*, and *p2k1-3* plants with ATP treatment over a time-course. Indeed, after ATP treatment, fluctuation in the expression level of *FPS1* in wild-type plants was significantly changed compared to mock-treated wild-type plants, whereas no difference in expression was observed in *mvk-1* mutants compared to *mvk-1* mock-treated plants (Fig. 7j). The level of *FPS1* was slightly reduced in *p2k1-3* plants after addition of ATP compared to *p2k1-3* mock-treated plants (Fig. 7j). Given the misregulation of defense-related genes through perturbation of *FPS1* expression, these results suggest that eATP, as a danger signal, impacts the expression of *FPS1* via *MVK*, likely through the direct action of P2K1 on MVK enzymatic activity.

## Discussion

Both in animals and plants, various intermediates and metabolites derived from the MVA pathway are known to play critical roles, including sterol synthesis, growth, defense response, and development^2,59^. Given that the products derived from the mevalonate pathway are extremely diverse including cellular signaling compounds^59,60^, it is not surprising that this pathway would be subject to tight regulation, including the ability to respond to changing environmental conditions, including abiotic and biotic stress. In general, plant plasma-membrane localized receptor-like kinases (RLK), modulate cellular metabolism indirectly, commonly through action on cytoplasmic kinases that subsequently impact various metabolic pathways^61^. Thus, it is somewhat unusual that P2K1 should directly modulate MVA pathway activity through direct interaction and phosphorylation of MVK. However, there is precedence for such a model. Specifically, previous research showed that the plasma membrane RLK NORK (also called MtDMI2 or LjSYMRK) directly regulates the MVA pathway via interaction with HMGR^60,62^, which impacts nuclear calcium spiking known to be critical for legume nodulation. These same authors also showed that MVP triggers nuclear calcium spiking, implying that the legume MVK may be required for legume nodulation signaling. At present, the cognate ligand for the DMI2 receptor is unknown. Given that previous research has shown that purine nucleotides can promote legume nodulation^63^, it is intriguing to postulate that eATP is also playing a role in legumes through modulation of MVA activity.

Eukaryotic *MVK* genes have been studied in yeast, ginkgo, rat and human^64–67^. In particular, human MVK has been well studied in relation to auto-immune diseases such as mevalonate kinase deficiency (MKD), and this led to the development and discovery of important drugs, such as anakinra and canakinumab^4–6^. Approximately 80 *MVK* genetic mutations are known to be associated with MKD disease^68^. However, surprisingly given the number of MVA pathway mutants available in *Arabidopsis*^22^, to our knowledge, no plant *mvk* mutants have been characterized previously.

Previous research clearly indicates that eATP is an important stress signaling compound in plants, which in *Arabidopsis* is recognized by the P2K1 receptor, perhaps in combination with the recently identified, closely related P2K2 receptor^34^. Details regarding the importance of eATP signaling in plants are emerging, including evidence that P2K1 contributes to plant defense against pathogens^33,50,51^, ATP mediated ROS signaling, JA levels and S-acylation^33,37,51^. We can now add to this list the ability of P2K1 to directly alter plant metabolism by regulation of MVK.

Identification of MVK as an interacting partner of P2K1 receptor is fundamentally important for understanding how plants modulate metabolic pathways through the RLK-triggered signaling pathway. Unlike the early secondary signaling messengers (Ca^2+^, ROS), it may be necessary to go through several signaling steps to regulate metabolism. Tripathi et al. showed that ATP treatment did not affect SA levels at an early time point, but showed that eATP did induce some SA-dependent genes^51^. Another study showed that ATP reduced SA levels at a later time point^69^. Interestingly, SA-related marker genes were strongly down-regulated in *p2k1-3* mutant plants 24 h after pathogen treatment (Fig. 7g–i). These results are consistent with our findings that ATP reduced SA levels, and reduction of SA-related gene expression (*ICS1*, *PR1*, and *PR2*), which we attribute to modulation of MVA pathway activity.

As mentioned above, research in *M. truncatula* showed that HMGR activity in the MVA pathway was essential for the ability of the invading rhizobium to induce the calcium oscillations necessary for successful nodulation^60^. This work is fully consistent with the results presented here showing a key role of the MVA pathway of mediating the cytoplasmic calcium response to elicitation, be it via eATP, rhizobia or pathogens. The question then is how does this metabolic pathway impact cellular responses that originate very quickly at the plasma membrane via the action of various RLKs. Current results do not provide a ready explanation for these observations. However, as a working hypothesis, it seems reasonable that reduction of the key MVA metabolic intermediates, such as IPP, involved in membrane anchoring of a variety of proteins, including receptors^12,70^, could drastically affect the overall environmental response of plants to any number of factors. In addition, reduction in the MVA pathway attenuates ATP-induced P2K1 accumulation and phosphorylation.

In conclusion, we identified *Arabidopsis* MVK as a direct phosphorylation target of P2K1, which results in activation of the MVA pathway in response to eATP elicitation, resulting in a variety of metabolic changes in the plant (Fig. 7k). Understanding this particular connection between purine signaling and the MVA pathway may ultimately provide potential molecular genetic targets for engineering crops with increased beneficial metabolites, or enhanced yield or improved stress resistance. The results also add to a growing body of evidence that purinergic signaling is as central in plants as it is in animals.

## Methods

### Plant materials and growth conditions

Wild-type aequorin-expressing transgenic *Arabidopsis* ColQ (Col-0 background) plants were kindly provided by Marc Knight^36^. The EMS-induced mutant population was described previously^30^. The *24-14* (subsequently referred to as *mvk-1*) mutant was backcrossed with the ColQ aequorin transgenic line three times (BC3F3). This backcrossed line was then used for phenotyping. *hmgr1-1* (Salk_125435) mutant was purchased from *Arabidopsis* Stock Center (http://www.arabidopsis.org/). *Arabidopsis* seeds were sown onto half strength Murashige and Skoog (MS) medium containing 1% (w/v) sucrose, 0.5% (w/v) phytagel, and 0.05% (w/v) MES pH 5.7. After 4 °C cold treatment for three days, the plates were placed vertically in a growth chamber (16 h light/8 h dark cycle, 22 °C, 100 μE cm^−2^ sec^−1^ light intensity). For other experiments, 10-day-old seedlings or 3-week-old plants were grown in PRO-MIX soil (Premier Tech Horticulture) in a growth chamber (16 h light/8 h dark cycle, 22 °C, 70% humidity and 150 μE cm^−2^ sec^−1^ light intensity).

### Plasmid constructs and plant/protoplast transformation

Full-length *MVK* (*At5g27450*) and *MKK3* (*At5g40440*) genes were amplified using gene-specific primers (Supplementary Table 1) and cDNA derived from wild-type plants. The PCR products were cloned into pDONR-Zeo (Invitrogen) or CloneJET (Thermo Fisher Scientific) vectors.

For constructs expressing recombinant proteins in *E. coli*, the cDNA of *MVK* and *MKK3* were ligated into pET21a (Novagen) resulting in a His-tag fusion at the C-terminus of the protein. The P2K1 kinase domain and P2K1-1 kinase-dead in pGEX-5X-1 were previously described^30^. For cloning the various restriction enzymes (Supplementary Table 1) and T4 ligase (Promega) were used. MVK-S77A, S78A, T79A, S87A, S89A, T169A, S208A, T222A, S263A, T285A, S329A, T342A, and MKK3-kinase dead clones were generated by site-directed mutagenesis.

For Bimolecular Fluorescence Complementation (BiFC) assay, full-length coding sequences of *MVK*, *P2K1*, and *MKK3* without stop codons were cloned into entry vector pDONR-Zeo and subcloned into pAM-PAT-35S::YFP, pAM-PAT-35S::YFPn, and pAM-PAT-35S::YFPc destination vectors through LR reaction^71^.

For co-immunoprecipitation assays in *Nicotiana benthamiana*, full-length CDS of *MVK* and *MKK3* from the pDONR-Zeo vectors were cloned into pGWB17, and full-length CDS of *P2K1* from the pDONR-Zeo vector was cloned into pGWB14 using LR reaction.

To generate constructs for the split-luciferase complementation assay in *N*. *benthamiana*, full-length CDS of *MVK*, *P2K1*, and *MKK3* from the pDONR-Zeo vectors were cloned into pCAMBIA-GW-Nluc and pCAMBIA-GW-Cluc using LR reaction.

In order to generate stable transgenic *Arabidopsis* plants, the *MVK* promoter, a 1350 bp region containing the 5′-UTR and encoding the first 17 amino acids, was cloned into pDONR-Zeo. To generate a fusion to the β-glucuronidase (GUS) reporter gene, the promoter region was subcloned into a plant binary vector pMDC162, generating the reporter construct *pMVK::GUS*.

The MVK promoter fragment (1350 bp, see above) was fused with the *MVK* gene, followed by cloning into the binary vectors pGWB13 using LR reaction. *MVK* gene mutant clones containing different, mutated phosphorylation sites were generated by site-directed mutagenesis. Those fragments were cloned into the pGWB13 vector using LR reaction. These binary vectors were transformed into *Agrobacterium tumefaciens* GV3101 and used for transformation of *Arabidopsis* plants via the floral dip method^72^. The homozygous T3 lines were screened based on hygromycin resistance.

To generate CRISPR/Cas9 constructs^73^, the Cas9 gene (UBQ3-Cas9-SK), driven by the UBQ3 promoter and the chimeric single guide RNA (AtU6–26-SK), driven by the AtU6–26 promoter was obtained from addgene (https://www.addgene.org/crispr/plant/). The *bar* gene, driven by mannopine synthase promoter of the binary vector pFGC5941, was used as a selection marker for *Arabidopsis* transformation. *MVK* specific single-guide RNA sequences were designed using the Zhang lab web-based tool: http://crispor.tefor.net/. Two gRNAs (MVK-253 and MVK-927) were used to create defined deletions within the exon of *MVK* gene. For each gRNA, a pair of DNA oligonucleotides (Supplementary Table 1) was synthesized and annealed to generate dimers. Subsequently, the annealed DNA was cloned using *Bbs*I restriction sites into pAtU6–26-SK to create pSK-AtU6–26-gRNA, and sequence integrity was confirmed by Sanger sequencing (University of Missouri, DNA Core Facility). To obtain a functional Cas9 expression construct for targeted mutagenesis, pSK-AtU6–26-gRNAs were cut with *Eco*RI-*Spe*I, and UBQ3-Cas9-SK was digested with *Sbf*I-*Spe*I. These 3 fragments were assembled into pFGC5941 by *Eco*RI-*Sbf*I restriction digestion followed by ligation to obtain pFGC5941-AtU6- UBQ3/Cas9 construct. All primers used in this study are listed in Supplementary Table 1.

### Cytoplasmic calcium assays

5-day-old seedlings were individually transferred to a single well of a 96-well plate with 50 μl of reconstitution buffer containing 10 μM coelenterazine (NanoLight Technology), 10 mM CaCl_2_ and 2 mM MES pH 5.7, and incubated overnight at room temperature in the dark. Fifty microliters of nucleotides, abiotic and biotic elicitor treatment solution (double concentration) were applied in each well. Aequorin bioluminescence imaging was performed using Photek CCD camera system (Photek 216; Photek, Ltd.). The camera was controlled by Photek PSU-1 intensifier and bioluminescence images were analysed using Photek/Image32 version 5.30. The production of luminescence (*L*) was monitored using an image-intensified CCD camera for 400 sec. One hundred microliters of discharging buffer containing 2 M CaCl_2_ and 20% (v/v) ethanol were used to estimate the remaining, unchelated aequorin, and the total luminescence (*L*_max_) was measured by the CCD camera for 400 sec. The calibration of calcium concentration levels was performed using the equation: pCa = 0.332588 × (−log *k*) + 5.5593, where *k* is a rate constant equal to luminescence photon counts (*L*) per second divided by total remaining photon counts (*L*_max_)^36^.

### EMS mutagenesis and mutant screening

In addition to the various *p2k1* mutants identified previously by screening an EMS-mutagenized library derived from *Arabidopsis thaliana* expressing aequorin^30^, an additional 7 mutants were identified whose mutations did not map to the *p2k1* gene. The *mvk-1* mutant line was among these seven, which led to the identification of the *mvk-1* mutant allele by map-based cloning and whole-genome sequencing. The response of the *mvk-1* mutant plants to ATP was tested by applying an increasing concentration (10, 100, 500, and 1000 μM) of ATP. We also tested the *mvk-1* plants for the specificity of their defect by applying a variety of known cytoplasmic calcium elicitors/treatments (e.g., 100 μM of ATP, ATPγS, ADP, ADPβS, AMP, Adenosine, GTP, ITP, CTP, TTP, and UTP; 100 nM of flg22, chitin, elf26, and pep1; 5 µM 3-OH-FAs; ice-cold water; 5% D-glucose; 300 mM NaCl and mannitol).

### Map-based cloning

We generated a mapping population by crossing the *mvk-1* mutant plants with *A. thaliana* Landsberg *erecta* ecotype plants, subsequently using the F2 generation for genotyping. The mutant phenotype was screened by monitoring the ATP-induced calcium response (via aequorin). Genetic mapping placed the *mvk-1* mutation on the short arm of chromosome 5 as measured by co-segregation with the ATP response phenotype. SSR marker nga76 and nga139 on chromosome 5 were strongly linked to the mutant phenotype and additional INDEL markers were used for further mapping. We narrowed the mutant phenotype region in the map to an ~146 kb interval between INDEL3576 (two recombinants in 76 F2 populations) and INDEL3630 (one recombinant in 76 F2 populations). All the molecular markers are listed in Supplementary Table 1.

### Whole genome sequencing

The *mvk-1* mutant plants were backcrossed three times with wild-type ColQ and homozygous lines (BC3F3) were obtained for whole genome sequencing. As an internal reference, DNA from the ColQ originally used for EMS mutagenesis was also prepared for sequencing. Single leaves from 75 plants (3 weeks-old) were pooled and genomic DNA extraction was performed according to the manufacturer’s instructions (Qiagen, DNeasy Plant Mini Kit). The whole genome sequencing was carried out by the DNA Core Facility of the University of Missouri (https://dnacore.missouri.edu/ngs.html). Genomic DNA (3 μg) was sheared to 350 bp and used for DNA PCR-Free library preparation. Sequencing was performed on a Hiseq 2000 (Illumina) instrument with 100 bp single end reads (>30× coverage for all DNA samples using 1 × 100 run). Reads were quality trimmed using FASTX FASTQ Quality Trimmomatic version 0.32^74^. Reads were then aligned back to the TAIR10 version of the *Arabidopsis* genome using Bowtie version 2^75^. Sam and output-pileup files were generated using Samtools version 0.1.7^76^. The output-pileup files converted to the NGM emap file in Next generation mapping web tool (http://142.150.215.220/ngm/) and then analyzed single nucleotide polymorphisms in Next-generation EMS mutation mapping website (http://142.150.215.220/ngm/cgi-bin/emap.cgi)^77^.

### MAPK phosphorylation assay

Leaf discs from 3-week-old plants were incubated in 2 mM MES pH 5.7 at room temperature overnight. After treatment with 100 µM ATP for 0, 5, 10, 30, and 60 min, total protein was extracted with extraction buffer containing 50 mM Tris-HCl pH 7.5, 150 mM NaCl, 1 mM EDTA, 0.5% Triton-X 100, 1 mM DTT, 0.1 mM PMSF, and 1X protein inhibitor (Pierce) for 1 h on ice. The extracted total proteins were mix with 5X Laemmli loading buffer containing 10% SDS, 50% glycerol, 0.01% bromophenol blue, 10% beta-mercaptoethanol, 0.3 M Tris-HCl pH 6.8, and heated in boiling water 5 min. The total extracted proteins were separated by 10% SDS-PAGE gel and detected by immunoblotting with rabbit anti-phospho-p44/p42 MAPK antibody (Cell signaling technology, Cat.No.50-191-932, dilution 1:1000) and secondary anti-rabbit-HRP (Sigma, Cat.No.12-348, dilution 1:10000).

### RNA isolation and quantitative real-time (qRT)-PCR

Pooled 10-day-old seedling plants (10 seedlings) were transferred into a 6-well plate and incubated in liquid MS medium in a growth chamber overnight. Samples were collected after treatment with 100 μM ATP.

For pathogen-induced gene expression test, 3-week-old leaves were treated with 2 mM MES (pH 5.7) and *P. syringae* DC3000 (OD_600_ = 0.002) for 24 h, then samples were collected.

Total RNA was extracted using a RNeasy Plant Mini Kit (Qiagen) according to the manufacturer’s instructions. RNA concentration was measured after Turbo DNA-free DNase (Ambion) treatment, and 1 μg RNA was used for first-strand cDNA synthesis using reverse transcriptase (Promega). The qPCR was performed using the PowerUp™ SYBR Green master mix (Applied Biosystems) following the manufacturer’s instructions. For data analysis, Delta Rn data were extracted from ABI 7500 PCR machine and LinReg software (version 11.0) was used to determine Cq data and baseline. Transcript levels were normalized against the expression of the *UBIQUITIN* (*UBQ*) or *SAND* (*At2g28390*) gene. The gene specific primers used are listed in Supplementary Table 1.

### MVK enzyme assay

2 μg of purified MVK-HIS protein was incubated with 2 μg GST-P2K1-KD kinase in reaction buffer (10 mM Tris-HCl pH 7.4, 5 mM MgCl_2_, 2 mM ATP, 100 μM Mevalonic acid). Reactions were kept at 30 °C for 10 min, and then terminated by adding an equal volume of acetonitrile. The samples were centrifuged for 20 min at 20,000 rcf and the supernatant was further processed for HPLC-MS/MS analysis. For quantification of mevalonic acid-5-phosphate, 50 μl internal standard working solution (containing 50 μM/L [13 C, 2H3]-DL mevalonic acid-5-phosphate) were added to 50 μl enzyme assay sample, calibrator or quality control sample. After vortexing, the samples were evaporated to dryness under a gentle stream of nitrogen. Subsequently, 50 μl butanol–HCl (Sigma–Aldrich) was added to derivatize the carboxy acid group of mevalonic acid-5-phosphate (Sigma–Aldrich) and its internal standard to the corresponding butyl ester at 70 °C for 45 min. The samples were centrifuged for 2 min at 1500 rcf and evaporated to dryness. The residue was dissolved in 1000 μl water/acetonitrile (1:1) and further diluted 1:100 with water/acetonitrile (1:1). The diluted solution was loaded and analyzed by a Waters Alliance 2695 high Performance liquid chromatography (HPLC) system coupled with Waters Acquity TQ triple quadrupole mass spectrometer (MS/MS). The analytes were separated by a Kinetex C18 (100 mm × 4.6 mm; 2.6 µm particle size) reverse-phase column (Phenomenex). The mobile phase consisted of 10 mM ammonium acetate and 0.1% formic acid in water (A) and 100% acetonitrile (B). The gradient conditions were 0–0.5 min, 2% B; 0.5–7 min, 2–80% B; 7.0–9.0 min, 80–98% B; 9.0–10.0 min, 2% B; 10.0–15.0 min, 2% B at a flow rate of 0.5 ml/min. The ion source in the MS/MS system was electrospray ionization (EI) operated in the negative ion mode [M-H]^−^ with capillary voltage of 1.5 kV. The ionization sources were programmed at 150 °C and the desolvation temperature was programmed at 450 °C. The MS/MS system was in the multi-reaction monitoring (MRM) mode with the optimized collision energy. The derivatized mevalonate-5-phosphate butyl ester was quantified with mass transition m/z 283→96.

### GUS staining and imaging

For detecting GUS activity, 10-day-old or 3-week-old plants were incubated in histochemical staining buffer (100 mM NaPO_4_ pH 7.0, 10 mM EDTA, 0.1% Triton X-100, 1 mM K_3_Fe(CN)_6_, 1 mM 5-Bromo-4-chloro-3-indoyl-beta-D-glucuronide) for 6 h and then washed with 70% EtOH until tissue cleared. For the wounding treatment, rosette leaves were crushed using a hemostat forceps.

### Co-immunoprecipitation assay

*Agrobacterium tumefaciens* GV3101 carrying the indicated constructs in infiltration buffer (10 mM MES pH 5.7, 10 mM MgCl_2_, 150 μM acetosyringone) was infiltrated into 3-week-old leaves of *N. benthamiana*. After 2 days, 200 μM of ATP and 2 mM MES (pH 5.7) were infiltrated into the same leaves. Total protein was extracted from pulverized (ground in liquid nitrogen) *N. benthamiana* leaf tissues using the following buffer: 50 mM Tris-HCl (pH 7.5), 250 mM NaCl, 10 mM MgCl_2_, 1 mM EDTA, 1 mM DTT, 0.2 mM PMSF, 10% glycerol, 0.5% Triton-X 100, and 1X protease inhibitor (Thermo Fisher Scientific) by gentle rotation at 4 °C for 2 h. The solution was centrifuged at 20,000 g for 10 min at 4 °C. The supernatant was transferred into a new tube and 1 μg anti-Myc-HRP (Santa Cruz, Cat.No.sc-40) was added, and incubated overnight with end-to-end shaking at 4 °C. Subsequently, 25 μl protein A resin (GenScript) was added for 4 h, spun down and washed five times with washing buffer containing 50 mM Tris-HCl pH 7.5, 150 mM NaCl, and 1X protease inhibitor. After washing, the resin was eluted with 50 μl 1X SDS-PAGE loading buffer and the eluent heated in boiling water for 10 min. The proteins were separated by 10% SDS-PAGE gel electrophoresis and detected by immunoblotting with both anti-HA-HRP (Roche, Cat. No.12 013 819 001, dilution 1:1000) and anti-Myc-HRP (Santa Cruz, Cat.No.sc-40, dilution 1:1000).

### Split-luciferase complementation imaging assay

*Agrobacterium tumefaciens* GV3101 containing the indicated constructs were incubated in infiltration buffer (10 mM MES pH 5.7, 10 mM MgCl_2_, 150 μM acetosyringone) for 2 h in dark and subsequently used to infiltrate into 3-week-old leaves of *N. benthamiana* for 2 days before the LUC activity measurement. 1 mM D-luciferin (Goldbio) was sprayed 1 time onto the leaves, and then kept in the dark for 7 min to allow the chlorophyll luminescence to decay, the luminescence was monitored using a CCD camera (Photek 216; Photek, Ltd.).

### Bimolecular fluorescence complementation assay

For the transient expression of split-YFP fused to P2K1, MVK or MKK3 proteins, the subcloned YFP protein fusion constructs were co-introduced by polyethylene glycol (PEG)-mediated transformation into *Arabidopsis* protoplasts prepared from leaf tissues of three- to four-week-old plants then incubated in a 23 °C growth chamber for 24 h under dark conditions^78^. The fluorescence signals were monitored using a Leica DM 5500B compound microscope with Leica DFC290 color digital camera equipped with various fluorescein filters 24 h after transformation. The fluorescence images were collected by the YFP (excitation wavelength of 514 nm) or FM4-64 (excitation wavelength of 558 nm) channels. Intact protoplasts without damage to the cell membrane were selected using a 20x objective lens in the bright field and the focus was adjusted. Then, the YFP signal was observed with the YFP filter under the same setting conditions. All images were taken with high magnification 63x oil immersion objective lens under the identical setting conditions. 5 μM FM4-64 dye (Thermo fisher scientific T13320) was used to stain the plasma membrane marker. This experiment was performed at the Molecular Cytology Core of the University of Missouri (https://research.missouri.edu/mcc/). Quantification of fluorescence intensity was performed with ImageJ software^79^.

### In vitro kinase assay

2 μg of purified GST, GST-P2K1-KD or GST-P2K1-1-KD kinase was incubated with 1 μg MVK-HIS (WT, S77A, S78A, T79A, S87A, S89A, T169A, S208A, T222A, S263A, T285A, S329A, T332A, and T342A) as substrate in a 20 μl reaction buffer (20 mM Tris-HCl pH 7.5, 10 mM MgCl_2_, 5 mM EGTA, 100 mM NaCl, and 1 mM DTT, 2 mM ATP, and 10 μCi radioactive [γ-^32^P] ATP) for 1 h at 30 °C. The reaction was stopped by boiling for 5 min with 5x SDS loading buffer. After electrophoresis in 12% SDS-PAGE, the gel was exposed for 12 h for autoradiography. The proteins within the gel were visualized with coomassie brilliant blue. Myelin basic protein (MBP) (Sigma), GST, and MKK3-KD were used as controls. Experiments were repeated independently three times.

### MVK phosphorylation site identification by LC-MS/MS

20 μg of purified GST-P2K1-KD kinase was incubated with 10 μg MVK-HIS as substrate in a 200 μl reaction buffer (20 mM Tris-HCl pH 7.5, 10 mM MgCl_2_, 5 mM EGTA, 100 mM NaCl, and 1 mM DTT, 2 mM ATP) for 30 min at 30 °C. In-solution trypsin digestion of the 25 μl reaction was performed at 37 °C O/N and 1 μg protein was loaded to identify phosphorylation sites by Bruker timsTOF Pro. A 40 min chromatographic separation (LC) was performed using Thermo C8 Pepmap 100 trap column and Bruker C18 (75 μm × 250 mm) of 1.6 μm particle size (OD4-25075 C18A) served as the analytical column. MS/MS were acquired within 100–1700 m/z using PASEF (Parallel Accumulation-Serial Fragmentation)^80^ 10 frames per 1.27 sec cycle (total 100–120 MS/MS). Automatic script (Bruker Data Analysis v5.1) was applied to convert RAW data to MGF file. Thermo Scientific Proteome Discoverer v 2.2 software running Sequest HT was used to search *Arabidopsis* proteome within NCBI database. Searches were conducted with tolerance of 50 ppm on the precursor and fragment ion mass tolerance of 0.1 dalton, and fixed modification of carbamidomethylation (C), variable modifications of oxidation (M) and phosphorylation (STY). Two biological replicate experiments were conducted. Obtained datasets are listed in Source data.

### Immunoblot assay

Total protein was extracted from 10-day-old *Arabidopsis* seedlings by homogenization in extraction buffer containing 50 mM Tris-HCl pH 7.5, 150 mM NaCl, 10 mM MgCl_2_, 1 mM EDTA, 1 mM DTT, 0.2 mM PMSF, 10% glycerol, 0.5% Triton-X 100, and 1X protease inhibitor (Sigma–Aldrich) for 4 h on ice. The samples were centrifuged at 20,000 rcf for 10 min at 4 °C, then the extracted total proteins were mix with 5X Laemmli loading buffer containing 10% SDS, 50% glycerol, 0.01% bromophenol blue, 10% beta-mercaptoethanol, 0.3 M Tris-HCl pH 6.8, and heated in boiling water 5 min. The total extracted proteins were separated by 12% SDS-PAGE gel and detected by immunoblotting with anti-HA-HRP (Roche, Cat. No.12 013 819 001, dilution 1:2000).

### Metabolomic analysis

Polar and semi-polar metabolites from 10-day-old *Arabidopsis* wild-type and *mvk-1* mutant roots were extracted following protocol^81^. Flash frozen pooled root samples were lyophilized and ground to a fine power with a tissue lyser. 800 µl of methanol/water (80:20) was added to 30 mg of pulverized root tissue, and samples were shaken at 1100 rpm for 1 h at 21 °C in a Thermomixer. Samples were centrifuged for 5 min and the supernatant was transferred to a new vial. All extracts were kept at −80 °C until metabolomic analyses.

Root extracts were analyzed with a high-resolution mass spectrometer (HRMS) Orbitrap Velos (Thermo Fisher Scientific) coupled to a Thermo Vanquish HPLC (Thermo Fisher Scientific) equipped with a heated electrospray ionization (HESI) source (Thermo Fisher Scientific). C18 (Hypersil Gold 150 × 2.1 mm, 3 µm particle size) reversed-phase column (Thermo Fisher Scientific) was used for the liquid chromatography at flow rate of 300 µl/min, while column compartment was maintained at 30 °C. HRMS was performed using Fourier transform mass spectrometry (FTMS) and full-scan mode at high resolving power (60,000 full width at half maximum). A mass range of 50–1000 m/z was acquired for each ionization mode. Samples were analyzed in both negative and positive ionization modes. Experimental blanks consisting of methanol/water (80:20) were injected every ~15 samples and used to determine the chromatographic background. A mixture of standards was analyzed every 40 samples to test for mass accuracy of the instrument and for further RT and m/z calibration purposes. One injection of methanol/water (80:20) was analyzed right after the standard mixture to avoid any alleged carry over and it was not used for background determination.

Obtained LC-MS RAW files were processed using MZmine 2 version 2.38^82^ (Supplementary Table 2) and a final dataset was exported to a CSV file (Supplementary Data 2). Using MZmine 2, metabolic features were assigned to specific metabolite identities using an in-house LC-MS library which contains over 600 typical metabolites from the primary and secondary metabolism of plants. Metabolite annotations were based on exact mass and retention time (RT) of the detected features corresponding to a second level of identification as detailed by the metabolomics standards initiative^83^. Metabolite annotation information for LC-MS data is detailed in Supplementary Data 3.

To ensure that interesting candidate metabolites were not thrown out during statistical analysis, peak intensity tables containing both previously annotated and unannotated peaks were submitted for missing data imputation using Metlmp1.2 (https://metabolomics.cc.hawaii.edu/software/MetImp/)^84^. Following missing data imputation, statistical analysis of the CSV files was done using MetaboAnalyst 4.0 (https://www.metaboanalyst.ca/)^85^. Data was normalized by median, log transformed, and pareto scaling was applied. Following data preparation, both volcano plot analysis (significance *P* < 0.05, FC > 1.5) and fold change analysis were carried out. Box-and-whisker plots were generated via MetaboAnalyst for further analysis. Fold change values were generated for the 7000+ peaks in the normalized data set. Fold change values for the same peak in the various treatments were grouped and analyzed for interesting patterns and flagged for identification.

### Bacterial inoculation assay

Three or four-week-old *Arabidopsis* seedlings were inoculated in 50 ml of *Pseudomonas syringae pv. tomato* DC3000 Lux (OD_600_ = 0.002) bacterial suspension (0.025% Silwet L-77 in sterile water) for 2 min at room temperature. After removing the bacterial solution, the plants were incubated in a growth chamber. To measure luminescence intensity, the first or second day after inoculation, bacterial growth was visualized and measured under a Photek CCD camera system (Photek 216; Photek, Ltd.). Then, the aerial part of plants of roughly the same size and fresh weight and sterilized with 70% ethanol for 1 min followed by a rinsing step with sterile water three times. The completely homogenized seedling tissue was diluted from 10^−2^ to 10^−6^ with 100 μl sterile water. 10 μl of serial dilutions were spotted onto King’s B agar plates containing rifampicin and kanamycin and the plates were incubated for 2 days. The bacterial colony forming units (CFU) were counted and analyzed in GraphPad Prism 7.

### Reporting summary

Further information on research design is available in the Nature Research Reporting Summary linked to this article.

## Supplementary information

Supplementary Information

Description of Additional Supplementary Files

Supplementary Data 1

Supplementary Data 2

Supplementary Data 3

Reporting Summary

## Data Availability

All data supporting the findings of this study are included in this manuscript and its supplementary files or further materials can be obtain from the corresponding author upon request. The BiFC microscopy and metabolites data are available via the following link (https://osf.io/5spkm/). The whole genome sequencing data that support the findings of this study have been deposited in NCBI Sequence Read Archive with the accession code PRJNA794855. Source data are provided with this paper.

## Source data

Source Data
