# Supplementary material for: Activation of the plant mevalonate pathway by extracellular ATP
Source: Nat Commun. 2022 Jan 21;13:450. doi: 10.1038/s41467-022-28150-w (PMC8783019; doi:10.1038/s41467-022-28150-w)
Supplement: Supplementary file 1 — Supplementary Information [file 41467_2022_28150_MOESM1_ESM.pdf]

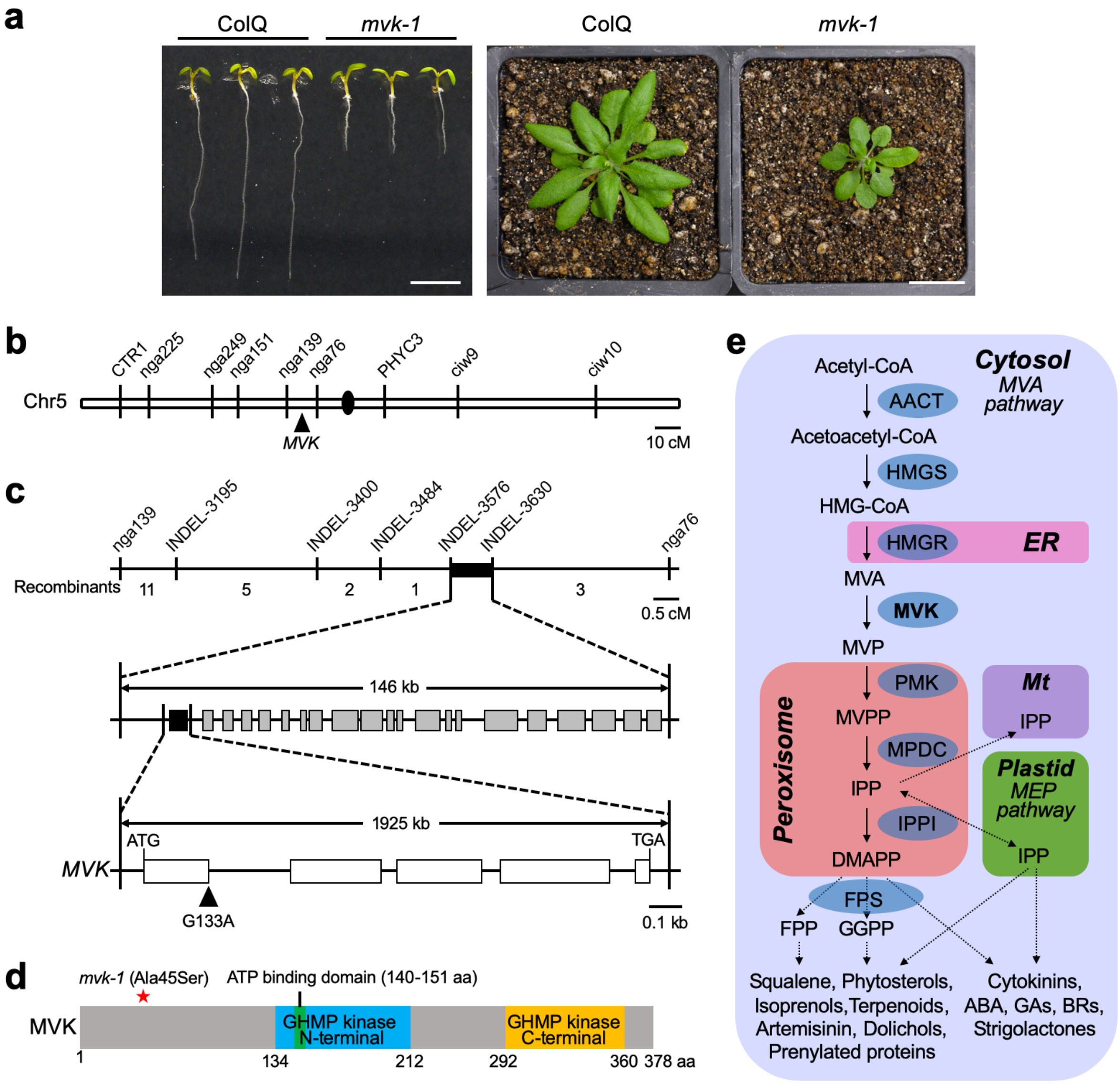

Supplementary Fig. 1 *mvk-1* mutant plants show reduced growth, and map-based cloning of *mvk-1* mutant

a 5-day-old (upper panel) and 5-week-old (bottom panel) seedlings of ColQ and *mvk-1* mutant plants. Scale bars: 0.5 cm (upper panel), 2 cm (bottom panel). **b** Map-based cloning of *mvk-1*. The *MVK* locus was mapped to a 9.6 Mb physical location between SSR markers nga139 and nga76 on the short-arm of chromosome 5. **c** SSR and INDEL markers were used to further delineate the 146 kb interval. ~2 kb segment showing Intron-Exon structure of the *MVK* gene. Illumina sequence analysis detected a single nucleotide substitution (i.e., G in wild-type to A in *mvk-1* mutant) in the first exon of the MVK ORF. **d** Domain structure of MVK. Red star indicates

amino acid substitution from Ala45 to Ser45 in *mvk-1*. Blue and orange boxes indicate Galactokinase, Homoserine kinase, Mevalonate kinase and Phosphomevalonate kinase (GHMP) N and C-terminal domain, respectively. Green box indicates ATP binding domain. **e** Simplified scheme of the isoprenoid biosynthetic pathways in plant cells. Figure redrafted from Vranová *et al*. 2013[22]. Solid-line arrows indicate a single enzymatic step, dashed line arrows indicate more than one enzymatic step. AACT, acetoacetyl-CoA thiolase; HMGS, 3-Hydroxy-3-Methylglutaryl-synthase; HMGR, 3-hydroxy-3-methylglutaryl-CoA reductase; MVK, mevalonate kinase; PMK, Phospho-Mevalonate kinase; MPDC, Diphospho-MVA decarboxylase; IPPI, Isopentenyl diphosphate isomerase; FPS, farnesyl diphosphate synthase; HMG-CoA, 3-Hydroxy-3-Methylglutaryl-CoA; MVA, Mevalonic acid; MVP, Mevalonic acid-5-phosphate; MVPP, Mevalonic acid-5-diphosphate; IPP, Isopentenyl diphosphate; DMAPP, Dimethylallyl diphosphate; FPP, farnesyl diphosphate; GGPP, geranylgeranyl diphosphate; ER, Endoplasmic reticulum; Mt, Mitochondria. ABA, abscisic acid; GAs, Gibberellic acids; BRs, Brassinosteroids.

a

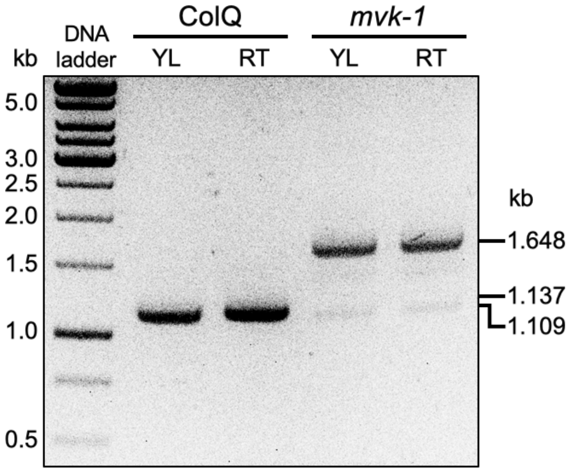

b Wild-type (1137 bp)

ATGGAAGTGAAAGCTAGAGCTCCTGGGAAGATCATACTTGCAGGGGAACACGCTGTTGTTCATGGATCCACCGCTGTAGCTGCCGCCATTGATCTCTACACTTA
CGTTACTCTCCGCTTTCCTCTTCCATCAGCTGAGAACAATGATAGGCTTACACTTCAGCTCAAGGACATTTCCTTGGAGTTTTCATGGTCCTTAGCCAGAATCA
AAGAAGCGATTCCTTATGATTCAAGCACTCTCTGCCGTTCTACGCCGGCTTCATGTTCAGAGGAGACCCTTAAATCAATTGCAGTTTTGGTTGAAGAGCAAAAT
CTTCCAAAGGAAAAGATGTGGCTCTCCTCTGGGATCTCCACGTTTCTCTGGTTATACACCAGAATTATAGGGTTCAATCCGGCTACAGTAGTCATTAACTCTGA
GCTTCCATACGGGTCTGGCCTCGGTTCATCAGCAGCTTTATGTGTAGCTCTCACAGCTGCTCTCCTTGCTTCTTCTATTTCAGAGAAAACCCGTGGTAACGGTT
GGTCATCTCTCGATGAAACCAATCTTGAGTTGCTAAATAAATGGGCTTTCGAAGGCGAAAAGATCATCCATGGGAAACCTTCTGGGATAGACAACACCGTCAGT
GCATACGGCAACATGATCAAGTTCTGCTCAGGCGAGATAACTCGGTTACAATCCAACATGCCTCTGAGAATGCTAATTACCAACACTAGAGTTGGGCGAAACAC
AAAAGCTCTGGTCTCTGGTGTGTCACAGAGAGCGGTAAGACATCCTGATGCGATGAAGTCAGTGTTCAACGCCGTGGATTCTATAAGCAAAGAGCTCGCTGCGA
TCATTCAGTCTAAAGACGAGACCTCAGTTACAGAAAAAGAAGAGAGAATAAAAGAACTCATGGAGATGAACCAAGGTCTGCTCCTGTCAATGGGGGTTAGCCAC
AGCTCAATCGAGGCTGTGATTCTAACCACGGTCAAGCACAAGCTTGTCTCCAAACTTACAGGAGCTGGTGGCGGCGGCTGCGTCCTCACTCTATTACCAACCGG
GACGGTGGTGGACAAAGTGGTGGAGGAGCTCGAGTCCAGCGGTTTTCAGTGTTTCACGGCATTGATTGGTGGTAACGGAGCTCAGATTTGCTATTGA

mvk-1 (1648 bp; premature stop codon)

ATGGAAGTGAAAGCTAGAGCTCCTGGGAAGATCATACTTGCAGGGGAACACGCTGTTGTTCATGGATCCACCGCTGTAGCTGCCGCCATTGATCTCTACACTTA
CGTTACTCTCCGCTTTCCTCTTCCATCAAgttcctttttttttccatattctgaggatttttttgtcttggtgttattgctactttgagatcaccaaacctgtcat
tactgtattgaactcatgagtgaattgtttagcaaaagatctgagctttttctaattcacttggggatatagcatatgcatgagtttacatttgttcaaatttg
gtttgggttctagactttgaatgttcatgttatgattgtctttgtgttagctaatgtcaccaagtaatagtattatcaaagggcttgaggctgtgttaagatgg
tagagtatcctgccttctatgtactacgggtggttaattcattctgcgctcgtgcttactagattcgagttcaaacagctaatgaaaatgtgatttcttaagtg
ccaataggcagatttgtagcaaatgacttgaccttattactgaattcaattcatgtttgattatactcttttgtcccatagaaacaaatctgaaagctttatgt
ttctttatttttttctgacagCTGAGAACAATGATAGGCTTACACTTCAGCTCAAGGACATTTCCTTGGAGTTTTCATGGTCCTTAGCCAGAATCAAAGAAGCGA
TTCCTTATGATTCAAGCACTCTCTGCCGTTCTACGCCGGCTTCATGTTCAGAGGAGACCCTTAAATCAATTGCAGTTTTGGTTGAAGAGCAAAATCTTCCAAAG
GAAAAGATGTGGCTCTCCTCTGGGATCTCCACGTTTCTCTGGTTATACACCAGAATTATAGGGTTCAATCCGGCTACAGTAGTCATTAACTCTGAGCTTCCATA
CGGGTCTGGCCTCGGTTCATCAGCAGCTTTATGTGTAGCTCTCACAGCTGCTCTCCTTGCTTCTTCTATTTCAGAGAAAACCCGTGGTAACGGTTGGTCATCTC
TCGATGAAACCAATCTTGAGTTGCTAAATAAATGGGCTTTCGAAGGCGAAAAGATCATCCATGGGAAACCTTCTGGGATAGACAACACCGTCAGTGCATACGGC
AACATGATCAAGTTCTGCTCAGGCGAGATAACTCGGTTACAATCCAACATGCCTCTGAGAATGCTAATTACCAACACTAGAGTTGGGCGAAACACAAAAGCTCT
GGTCTCTGGTGTGTCACAGAGAGCGGTAAGACATCCTGATGCGATGAAGTCAGTGTTCAACGCCGTGGATTCTATAAGCAAAGAGCTCGCTGCGATCATTCAGT
CTAAAGACGAGACCTCAGTTACAGAAAAAGAAGAGAGAATAAAAGAACTCATGGAGATGAACCAAGGTCTGCTCCTGTCAATGGGGGTTAGCCACAGCTCAATC
GAGGCTGTGATTCTAACCACGGTCAAGCACAAGCTTGTCTCCAAACTTACAGGAGCTGGTGGCGGCGGCTGCGTCCTCACTCTATTACCAACCGGGACGGTGGT
GGACAAAGTGGTGGAGGAGCTCGAGTCCAGCGGTTTTCAGTGTTTCACGGCATTGATTGGTGGTAACGGAGCTCAGATTTGCTATTGA

mvk-1 (1109 bp; premature stop codon)

ATGGAAGTGAAAGCTAGAGCTCCTGGGAAGATCATACTTGCAGGGGAACACGCTGTTGTTCATGGATCCACCGCTGTAGCTGCCGCCATTGATCTCTACACTTA
C~~GTTACTCTCCGCTTTCCTCTTCCATCAA~~CTGAGAACAATGATAGGCTTACACTTCAGCTCAAGGACATTTCCTTGGAGTTTTCATGGTCCTTAGCCAGAATCA
AAGAAGCGATTCCTTATGATTCAAGCACTCTCTGCCGTTCTACGCCGGCTTCATGTTCAGAGGAGACCCTTAAATCAATTGCAGTTTTGGTTGAAGAGCAAAAT
CTTCCAAAGGAAAAGATGTGGCTCTCCTCTGGGATCTCCACGTTTCTCTGGTTATACACCAGAATTATAGGGTTCAATCCGGCTACAGTAGTCATTAACTCTGA
GCTTCCATACGGGTCTGGCCTCGGTTCATCAGCAGCTTTATGTGTAGCTCTCACAGCTGCTCTCCTTGCTTCTTCTATTTCAGAGAAAACCCGTGGTAACGGTT
GGTCATCTCTCGATGAAACCAATCTTGAGTTGCTAAATAAATGGGCTTTCGAAGGCGAAAAGATCATCCATGGGAAACCTTCTGGGATAGACAACACCGTCAGT
GCATACGGCAACATGATCAAGTTCTGCTCAGGCGAGATAACTCGGTTACAATCCAACATGCCTCTGAGAATGCTAATTACCAACACTAGAGTTGGGCGAAACAC
AAAAGCTCTGGTCTCTGGTGTGTCACAGAGAGCGGTAAGACATCCTGATGCGATGAAGTCAGTGTTCAACGCCGTGGATTCTATAAGCAAAGAGCTCGCTGCGA
TCATTCAGTCTAAAGACGAGACCTCAGTTACAGAAAAAGAAGAGAGAATAAAAGAACTCATGGAGATGAACCAAGGTCTGCTCCTGTCAATGGGGGTTAGCCAC
AGCTCAATCGAGGCTGTGATTCTAACCACGGTCAAGCACAAGCTTGTCTCCAAACTTACAGGAGCTGGTGGCGGCGGCTGCGTCCTCACTCTATTACCAACCGG
GACGGTGGTGGACAAAGTGGTGGAGGAGCTCGAGTCCAGCGGTTTTCAGTGTTTCACGGCATTGATTGGTGGTAACGGAGCTCAGATTTGCTATTGA

Supplementary Fig. 2 Sequencing information of *mvk-1* mutant

a Splicing error in the *MVK* gene transcripts. Substitution mutation of *MVK* results in a splicing error in the *mvk-1* mutant. Difference in size of *MVK* cDNA in ColQ (1137 bp) and *mvk-1* (1648 bp and 1109 bp) mutant. YL, young leaf; RT, root. Experiment was repeated three times with similar results. **b** Wild-type and *mvk-1* coding sequences. *mvk-1* mutation causes splicing errors

in *MVK* transcript. Black letters indicate exon, orange letters indicate intron, and blue letter indicates mutated sequences. Underlined red letters indicate stop codon sequence. Green letters with middle-line indicate deleted sequences.

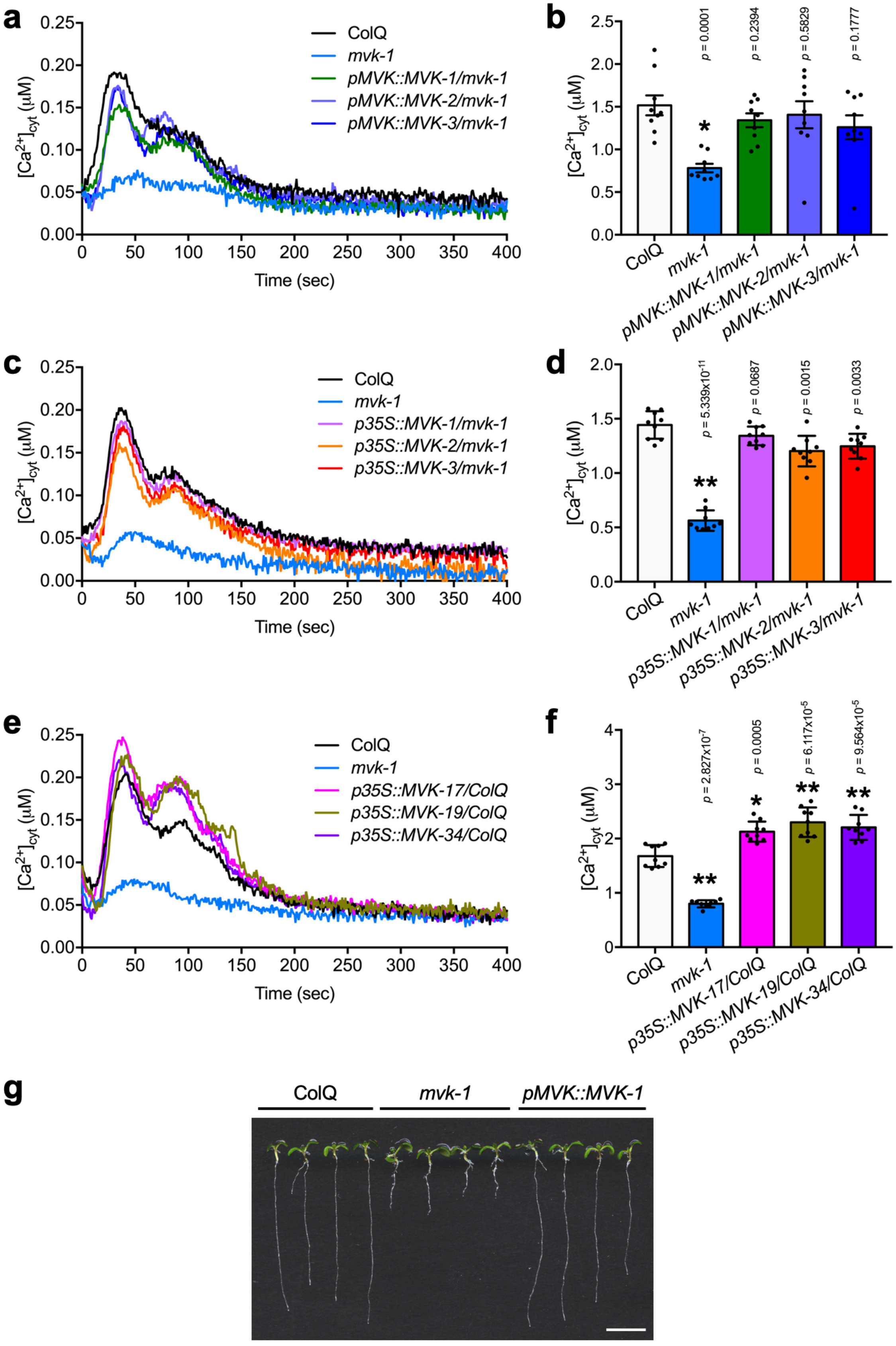
a
[Ca2+]cyt (μM)
Time (sec)
ColQ
mvk-1
pMVK::MVK-1/mvk-1
pMVK::MVK-2/mvk-1
pMVK::MVK-3/mvk-1
b
p = 0.0001
p = 0.2394
p = 0.5829
p = 0.1777
ColQ
mvk-1
pMVK::MVK-1/mvk-1
pMVK::MVK-2/mvk-1
pMVK::MVK-3/mvk-1
c
ColQ
mvk-1
p35S::MVK-1/mvk-1
p35S::MVK-2/mvk-1
p35S::MVK-3/mvk-1
d
p = 5.339x10-11
p = 0.0687
p = 0.0015
p = 0.0033
e
ColQ
mvk-1
p35S::MVK-17/ColQ
p35S::MVK-19/ColQ
p35S::MVK-34/ColQ
f
p = 2.827x10-7
p = 0.0005
p = 6.117x10-5
p = 9.564x10-5
g
ColQ
mvk-1
pMVK::MVK-1

Supplementary Fig. 3 The *mvk-1* mutant phenotype is complemented by expressing wild-type *MVK*

a, **c**, **e** Kinetics of intracellular calcium elevation after treatment with 100 μM ATP for 400 seconds. Experiment was repeated three times with similar results. **b**, **d**, **f** Comparison of the calcium response in the *mvk-1* mutant (blue bar) to those expressing the wild-type *MVK* gene under native (**a** and **b**, *mvk-1* background; *pMVK::MVK-1* to *3* are the independent transgenic lines in **a** and **b**) and *CaMV 35S* promoter (**c** and **d**, *mvk-1* background; *p35S::MVK-1* to *3* are the independent transgenic lines in **c** and **d**; **e** and **f**, ColQ background; *p35S::MVK-17*, *19*, and *34* are the independent transgenic lines in **e** and **f**), which resulted in recovered calcium levels in response to ATP. All data represented as mean ± SEM, n=9 seedlings, ($^{*}P < 0.001$, $^{**}P < 0.0001$, two-sided Student's *t*-test). Experiment was repeated three times with similar results. **g** 5-day-old seedlings of ColQ, *mvk-1*, and complemented line *pMVK::MVK-1* (wild-type *MVK* gene under the native promoter in *mvk-1* background). Scale bar: 0.5 cm.

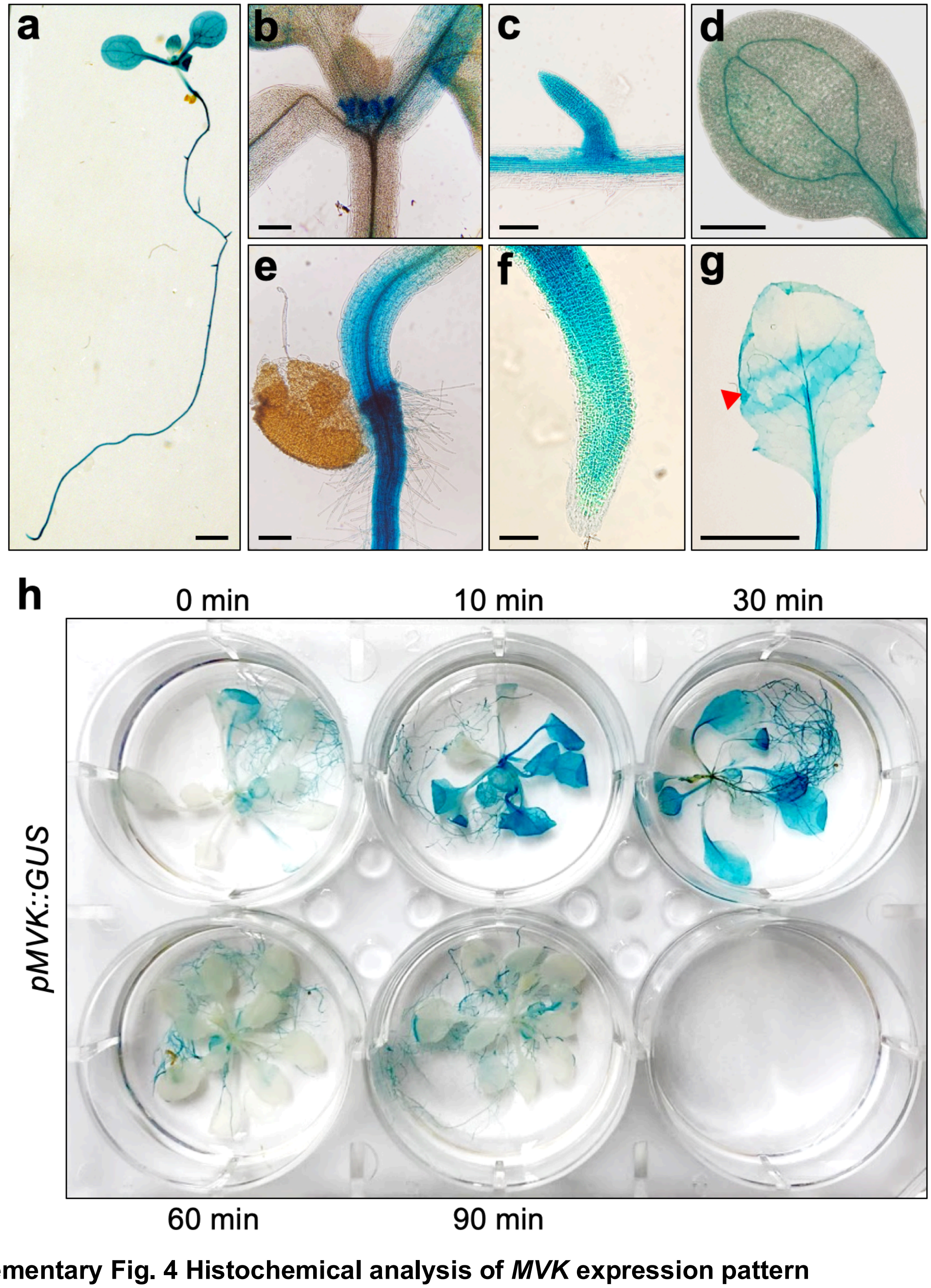

Supplementary Fig. 4 Histochemical analysis of *MVK* expression pattern

a-**g** Wild-type *Arabidopsis* plants transformed with a chimeric *pMVK::GUS* including 1.45 kb of the 5′ region of the *MVK* gene fused to the *GUS* coding sequence. **a** 10-day old seedling including the first pair of basal leaves. **b** 5-day-old seedling including the shoot apical meristem. **c** Lateral root of ten-day old seedling. **d** Second leaf of 5-day-old seedling. **e** Root apical

meristem of 5-day old seedling. **f** Root tip of 5-day-old seedling. **g** Wounding (red arrowhead) by hemostat forceps of two-week-old plant leaf. **h** ATP induced *MVK* expression in *pMVK::GUS* transgenic plants. 3-week-old plants were treated with 100 μM ATP for 0, 10, 30, 60, and 90 minutes and incubated with X-gluc (25 mg/ml) for 3 hours at room temperature. Scale bars: 2 mm (a), 0.5 mm (b, c, e, f), 1 mm (d), 1 cm (g).

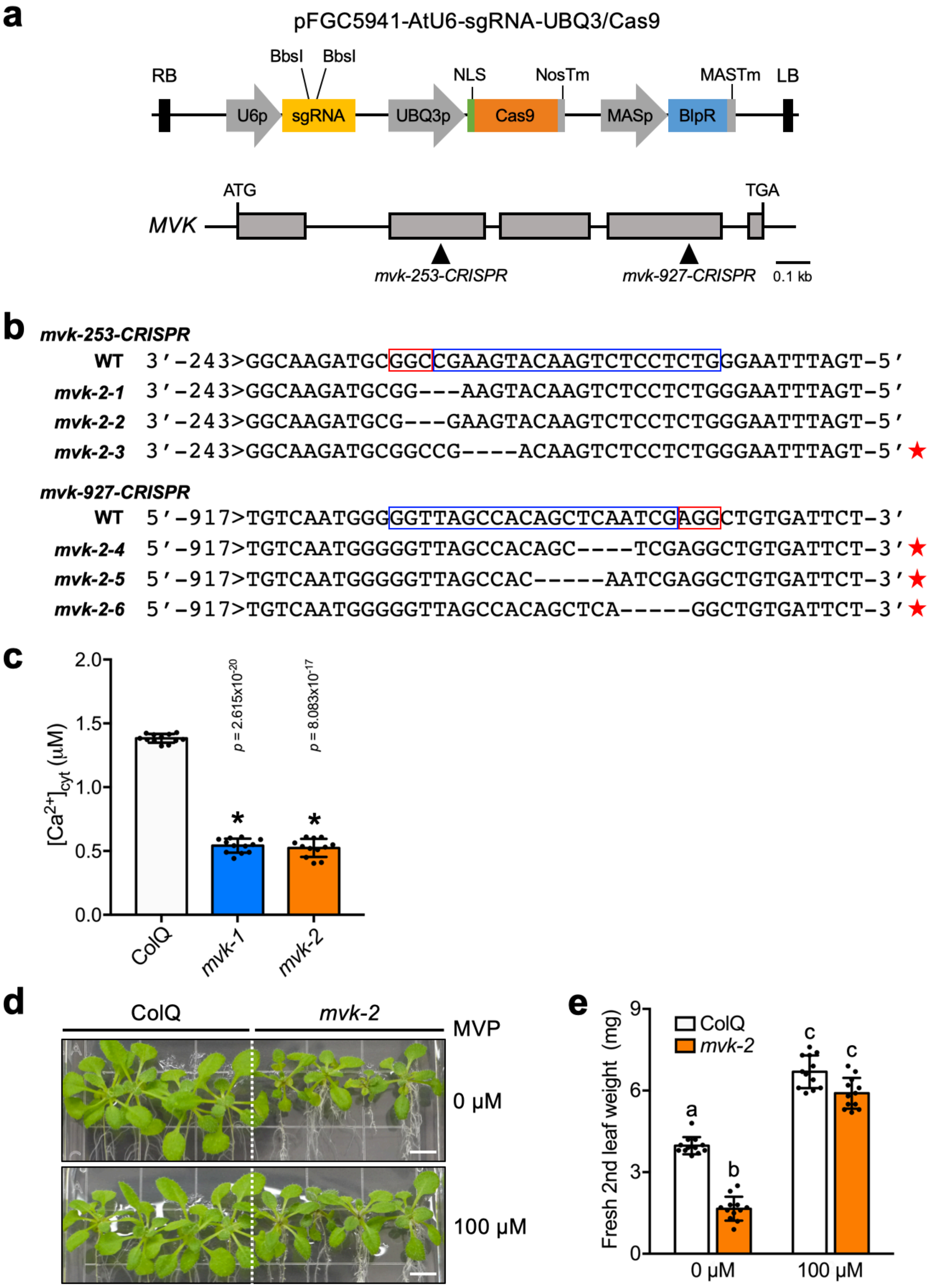
a
pFGC5941-AtU6-sgRNA-UBQ3/Cas9
RB
BbsI BbsI
U6p
sgRNA
UBQ3p
NLS
Cas9
NosTm
MASp
BlpR
MASTm
LB
ATG
TGA
MVK
mvk-253-CRISPR
mvk-927-CRISPR
0.1 kb
b
mvk-253-CRISPR
WT 3'-243>GGCAAGATGCGGCCGAAGTACAAGTCTCCTCTGGGAATTTAGT-5'
mvk-2-1 3'-243>GGCAAGATGCGG---AAGTACAAGTCTCCTCTGGGAATTTAGT-5'
mvk-2-2 3'-243>GGCAAGATGCG---GAAGTACAAGTCTCCTCTGGGAATTTAGT-5'
mvk-2-3 3'-243>GGCAAGATGCGGCCG----ACAAGTCTCCTCTGGGAATTTAGT-5'
mvk-927-CRISPR
WT 5'-917>TGTCAATGGGGGTTAGCCACAGCTCAATCGAGGCTGTGATTCT-3'
mvk-2-4 5'-917>TGTCAATGGGGGTTAGCCACAGC----TCGAGGCTGTGATTCT-3'
mvk-2-5 5'-917>TGTCAATGGGGGTTAGCCAC-----AATCGAGGCTGTGATTCT-3'
mvk-2-6 5'-917>TGTCAATGGGGGTTAGCCACAGCTCA-----GGCTGTGATTCT-3'
c
[Ca2+]cyt (μM)
2.0
1.5
1.0
0.5
0.0
p = 2.615x10-20
p = 8.083x10-17
ColQ
mvk-1
mvk-2
d
ColQ
mvk-2
MVP
0 μM
100 μM
e
Fresh 2nd leaf weight (mg)
9
6
3
0
ColQ
mvk-2
a
b
c
c
0 μM
100 μM

Supplementary Fig. 5 *mvk* CRISPR/Cas9 mutants, and their Ca^{2+} and exogenous MVP responses

a Schematic structure of the CRISPR/Cas9 vectors used in this study. The sgRNA cassette including the 20-base-pair target site was amplified and integrated into pFGC5941-Cas9. The resulting vector contains the sgRNA cassette as well as the *Cas9* gene under the control of the *UBIQUITIN3* promoter (UBQ3p), and the BlpR (bialophos or phosphinothricine) resistance gene as selection marker. RB, right T-DNA border; U6p, U6-26 promoter; sgRNA, single guide RNA; NLS, nuclear localization signal; NosTm, nopaline synthase terminator; MASp, MAS promoter; MASTm, MAS terminator; LB, left T-DNA border. **b** Various types of CRISPR/Cas9-induced mutations detected by amplicon sequencing in T1 plants. The blue line boxes indicate target sites and red line boxes are PAM sequences. *mvk-2-1* to *mvk-2-6* are the individual T1 lines for the transgenic plants. Some mutations from the different mutant lines generate a premature stop codon (Red star). **c** The bar graph shows the integrated, cytoplasmic calcium response to 100 μM ATP in both *mvk-1* and *mvk-2* (*mvk-2-4* line) mutants. Data are shown as mean ± SEM, n=12 seedlings ($^{*}P < 0.01$, two-side Student's *t*-test). Experiment was repeated three times with similar results. **d** 16-day-old ColQ and *mvk-2* (*mvk-2-4* line) mutant seedlings grown on medium without or with 100 μM mevalonic acid-5-phosphate (MVP). Scale bars: 0.5 cm. **e** Fresh second leaf weight of 16-day-old plants [n=12 seedlings for ColQ and n=12 seedlings for *mvk-2* (*mvk-2-4* line)]. Data represent mean ± SEM from independent experiments. One-way ANOVA analysis was calculated by GraphPad Prism 7. Means with different letters are significantly different ($P < 0.05$). *P*-values indicate significance relative to ColQ with mock treatment and were determined by one-sided ANOVA with multiple comparisons and adjusted using a Duncan post hoc test. Experiment was repeated three times with similar results.

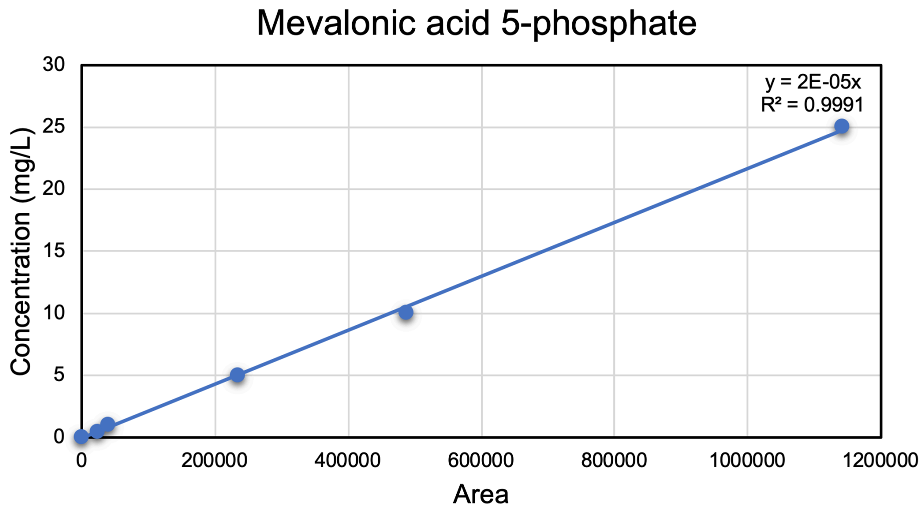

Supplementary Fig. 6 Linear range of the mevalonic acid 5-phosphate concentrations

The ion chromatogram of the linear range of mevalonic acid 5-phosphate (MVP) was quantified by LC-MS/MS analysis. This range covers from 0.05 mg/L to 25 ppm that allow us to capture any product formation in this linear range.

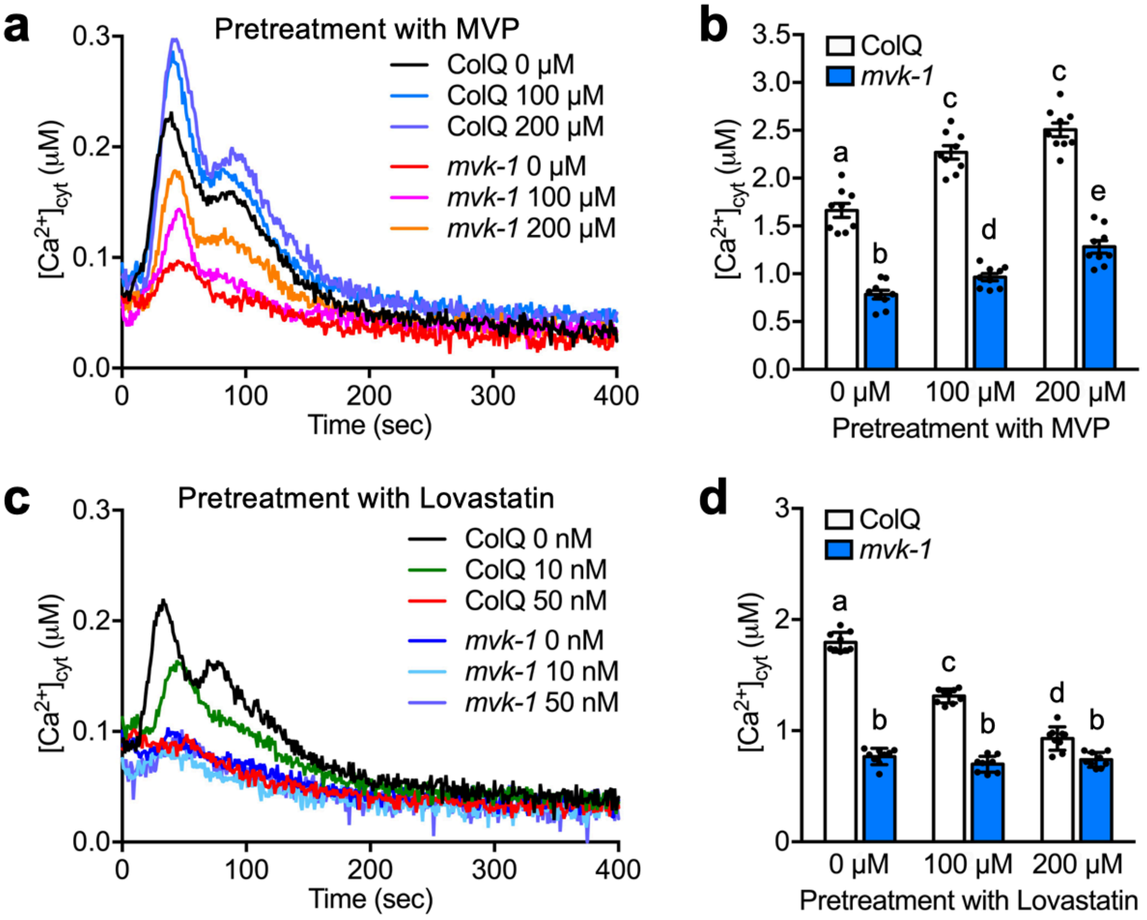

Supplementary Fig. 7 exogenous application of mevalonic acid-5-phosphate (MVP) or lovastatin affect ATP-triggered calcium production

a The kinetics of the calcium response to 100 μM ATP. 5-day-old ColQ and *mvk-1* seedlings were pre-incubated for 1 hour with 0, 100, or 200 μM MVP. **b** The bar graph shows the integrated calcium concentration in **a** for 400 seconds in both ColQ and *mvk-1* mutants (n=9 seedlings). **c** The kinetics of the calcium concentration in responses to 100 μM ATP. 5-day-old ColQ and *mvk-1* seedlings were pre-incubated for 1 hour with 0, 10, or 50 nM lovastatin. **d** The bar graph shows the integrated calcium concentration in **c** for 400 seconds in both ColQ and *mvk-1* mutants (n=9 seedlings). Data represent mean ± SEM from independent experiments. Means with different letters are significantly different ($P < 0.05$). *P*-values indicate significance relative to ColQ with mock treatment and were determined by one-sided ANOVA with multiple comparisons and adjusted using a Duncan post hoc test. All above experiments were repeated three times with similar results.

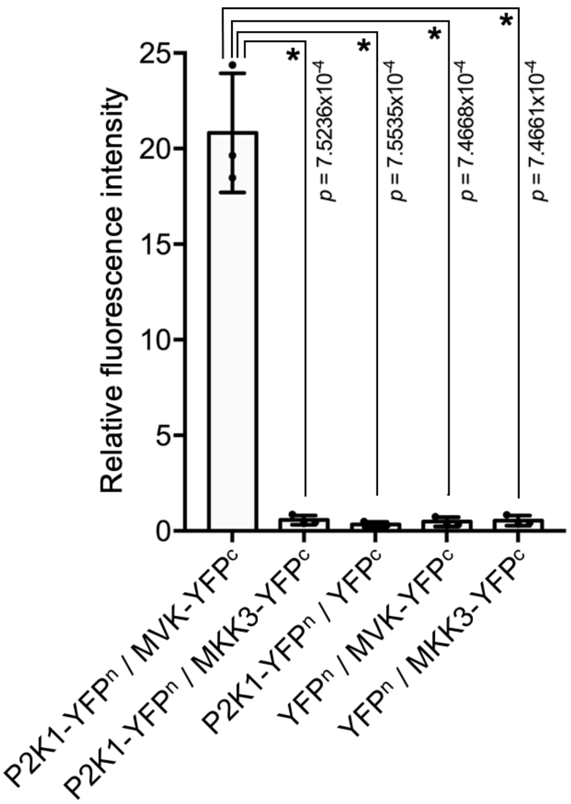

Supplementary Fig. 8 Quantification of relative fluorescence intensity of P2K1-MVK Biomolecular fluorescence complementation (BiFC) system

The indicated constructs were transiently expressed in wild-type protoplasts and the BiFC assay was performed. Relative fluorescence intensity was measured by ImageJ software. The asterisks indicate statistical significance (n=3 protoplasts, $^{*}P < 0.001$, two-sided Student's *t*-test). Experiment was repeated three times with similar results.

a

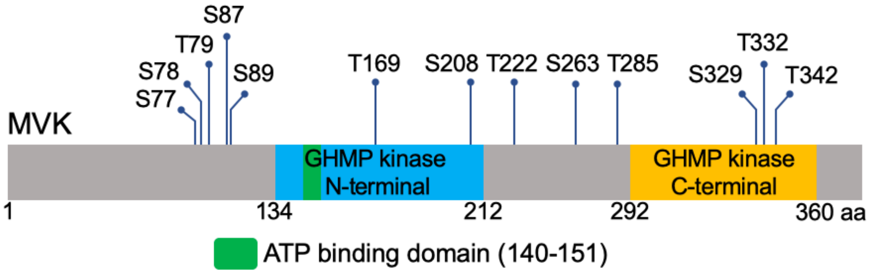

	Phosphorylation sites
Serine (S)	S77, S78, S87, S89, S208, S263, S329
Threonine (T)	T79, T169, T222, T285, T332, T342

```
1          11         21         31         41         51         61
MEVKARAPGK IILAGEHAVV HGSTAVAAAI DLYTYVTLRF PLPSAENNDR LTLQLKDISL EFSWSLARIK

71         81         91         101        111        121        131
EAIPYDSSTL CRSTPASCSE ETLKSIAVLV EEQNLPKEKM WLSSGISTFL WLYTRIIGFN PATVVINSEL

141        151        161        171        181        191        201
PYGSGLGSSA ALCVALTAAL LASSISEKTR GNGWSSLDET NLELLNKWAF EGEKIIHGKP SGIDNTVSAY

211        221        231        241        251        261        271
GNMIKFCSGE ITRLQSNMPL RMLITNTRVG RNTKALVSGV SQRAVRHPDA MKSVFNAVDS ISKELAAIIQ

281        291        301        311        321        331        341
SKDETSVTEK EERIKELMEM NQGLLLSMGV SHSSIEAVIL TTVKHKLVSK LTGAGGGGCV LTLLPTGTVV

351        361        371
DKVVEELESS GFQCFTALIG GNGAQICY
```

b

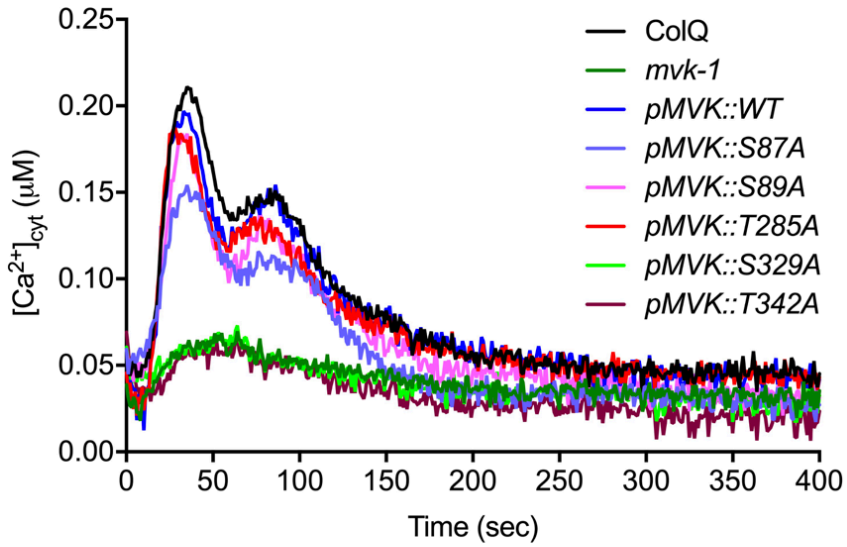

Supplementary Fig. 9 Mapping of Mevalonate kinase phosphorylation sites by LC-MS/MS and ATP-triggered calcium kinetics in wild-type, *mvk-1*, and MVK phospho-mutants

a Schematic representation of MVK protein structure highlighting P2K1-mediated MVK phosphorylation sites identified by mass spectrometry. P2K1 mediated phosphorylation residues are indicated in red. See also total dataset shown in Source data file. **b** The kinetics of the

cytoplasmic calcium response to 100 µM ATP for 400 seconds in 5-day-old ColQ, *mvk-1*, and MVK wild-type or phospho mutants (n=9 seedlings). The indicated constructs were expressed in the *mvk-1* mutant background. All above experiments were repeated three times with similar results.

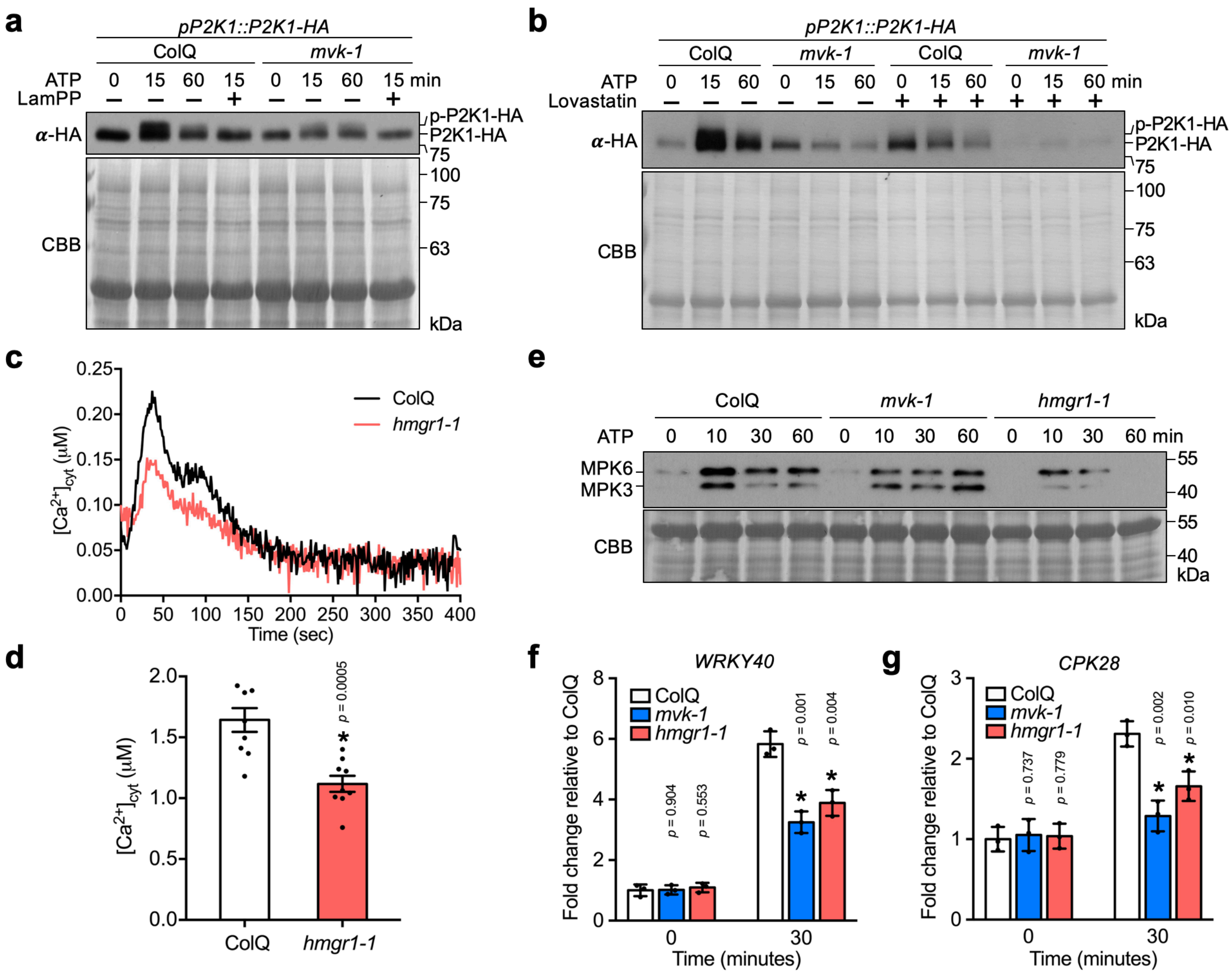

Supplementary Fig. 10 *mvk-1* mutant shows reduced P2K1 and ATP-induced P2K1 phosphorylation. *hmgr1-1* mutant shows lower ATP-induced cytoplasmic calcium influx, MPK3/6 phosphorylation, and ATP-induced gene expression

a,b MVK regulates P2K1 accumulation and phosphorylation. P2K1-HA protein was detected by anti-HA immunoblot in 10-day-old ColQ and *mvk-1* mutant backgrounds in response to 200 μM of ATP over a time-course from 0 to 60 min. Lambda protein phosphatase was used to dephosphorylate P2K1. 10-day-old ColQ and *mvk-1* plants were pre-treated with 2 mM MES (pH 5.7) (mock) and 100 nM lovastatin for 24 hours before ATP treatment. p-P2K1-HA: phosphorylated P2K1-HA. LamPP: Lambda protein phosphatase. Coomassie brilliant blue (CBB) staining (bottom panel) showed equal loading. **c** The kinetics of the cytoplasmic calcium response to 100 μM ATP for 400 seconds in ColQ and *hmgr1-1* mutant. **d** The bar graph shows the integrated calcium response to 100 μM of ATP for 400 seconds in ColQ and *hmgr1-1*

mutant. Asterisks indicate significant differences between ColQ and *hmgr1-1* mutant (means ± SEM, n=9 seedlings, $^{*}P < 0.001$, two-sided Student's *t*-test). **e** *hmgr1* mutant exhibits reduced phosphorylation of MPK3 and MPK6 in response to 100 µM of ATP compared to wild-type over a time-course from 0 to 60 min. Phosphorylation of MPK3 and MPK6 was detected using antibody against phospho-p44/p42 mitogen-activated protein kinase. *mvk-1* mutant was used as a negative control. Coomassie brilliant blue (CBB) staining (bottom panel) showed equal loading. **f, g** Relative expression of *WKRY40* and *CPK28* in 10-day-old ColQ, *mvk-1*, and *hmgr1-1* whole seedlings treated with 100 µM ATP for 30 minutes was performed using qRT-PCR analysis. Gene expression data were normalized using the *SAND* reference gene. The bar graphs are means of three biological repeats. Asterisks indicate the significant differences compared to ColQ at the same time points ($^{*}P < 0.05$, two-sided Student's *t*-test). All above experiments were repeated three times with similar results.

Supplementary Table 1. Sequence of primers used in this study

Name	5'-sequence-3'	Objective
CTR1-F	CCACTTGTTTCTCTCTCTAG	Map-based cloning
CTR1-F	TATCAACAGAAACGCACCGAG	
nga76-F	AGGCATGGGAGACATTTACG	
nga76-R	GGAGAAAATGTCACTCTCCACC	
nga139-F	GGTTTCGTTTCACTATCCAGG	
nga139-R	AGAGCTACCAGATCCGATGG	
nga151-F	CAGTCTAAAAGCGAGAGTATGATG	
nga151-R	GTTTTGGGAAGTTTTGCTGG	
nga225-F	TCTCCCCACTAGTTTTGTGTCC	
nga225-R	GAAATCCAAATCCCAGAGAGG	
nga249-F	GGATCCCTAACTGTAAAATCCC	
nga249-F	TACCGTCAATTTCATCGCC	
PHYC.3-F	AAACTCGAGAGTTTTGTCTAGATC	
PHYC.3-R	CTCAGAGAATTCCCAGAAAAATCT	
ciw9-F	CAGACGTATCAAATGACAAATG	
ciw9-R	GACTACTGCTCAAACTATTCGG	
ciw10-F	CCACATTTTCCTTCTTTCATA	
ciw10-R	CAACATTTAGCAAATCAACTT	
UPSC3195-F	GCGATACATTCAAATGTAA	
UPSC3195-R	CCTATCGGATCAGCTATCAC	
UPSC3400-F	TATTCGTTTTCTGAACATGA	
UPSC3400-R	AAACACACCACCATTTAAAG	
UPSC3484-F	GGATAACTTCGAAGCCAACT	
UPSC3484-R	ATAGTCCTGTCTTGTGTCA	
UPSC3576-F	CAATTGAGCCAAAACTTAAC	
UPSC3576-R	AATTCAATTCTTCGGTGTAG	
UPSC3630-F	AAACCCCACCGGAAAACC	
UPSC3630-R	ATAATCACGAAAAACATGAC	
MVK-F	GGGGACAAGTTTGTACAAAAAAGCAGGCTTCATGGAAGTGAAAGCTAGAGCT	pDONR-Zeo-MVK
MVK(no stop)-R	GGGGACCACTTTGTACAAGAAAGCTGGGTCATAGCAAATCTGAGCTCCGTT	
MVK(stop)-R	GGGGACCACTTTGTACAAGAAAGCTGGGTCTCAATAGCAAATCTGAGCTCCGTT	
MVK-EcoRI-F	GGAATTCATGGAAGTGAAAGCTAGAGCT	pET21a-MVK-HIS
MVK(stop)-SalI-R	GGTCGACTCAATAGCAAATCTGAGCTCC	
MVK(no stop)-SalI-R	GGTCGACATAGCAAATCTGAGCTCC	
MKK3-F	GGGGACAAGTTTGTACAAAAAAGCAGGCTTCATGGCGGCATTGGAGGAGCTAAAG	pDONR-Zeo-MKK3
MKK3(no stop)-R	GGGGACCACTTTGTACAAGAAAGCTGGGTGATCTAAGTTTGTAATATAAAGCTCTTGC	
MKK3-SalI-F	GTCGACGGATGGCGGCATTGGAGGA	pET21a-MKK3-HIS
MKK3(no stop)-XhoI-R	CTCGAGATCTAAGTTTGTAATAT	
MKK3-K112M-F	CATAGAATTTTAGCGTTGATGAAGATTAATATCTTTGAAA	pET21a-MKK3-HIS kinase dead
MKK3-K112M-R	TTTCAAAGATATTAATCTTCATCAACGCTAAAATTCTA	

	TG	
proMVK-F	GGGGACAAGTTTGTACAAAAAAGCAGGCTAACTCT TGAAGCTAACTTCAC	pDONR-Zeo-proMVK
proMVK-R	GGGGACCACTTTGTACAAGAAAGCTGGGTGTGTTC CCCTGCAAGTATGAT	
MVK-S77A-F	TATGATgCAAGCACTCTCTGCCG	site-directed mutagenesis
MVK-S77A-R	AGTGCTTGcATCATAAGGAATCG	
MVK-S78A-F	GATTCAgcCACTCTCTGCCGTTC	
MVK-S78A-R	GAGAGTGgcTGAATCATAAGGAA	
MVK-T79A-F	TCAAGCgCTCTCTGCCGTTCTAC	
MVK-T79A-R	GCAGAGAGcGCTTGAATCATAAG	
MVK-S87A-F	CCGGCTgCATGTTCAGAGGAGAC	
MVK-S87A-R	TGAACATGcAGCCGGCGTAGAAC	
MVK-S89A-F	TCATGTgCAGAGGAGACCCTTAA	
MVK-S89A-R	CTCCTCTGcACATGAAGCCGGCG	
MVK-T169A-F	GAGAAAgCCCGTGGTAACGGTTG	
MVK-T169A-R	ACCACGGGcTTTCTCTGAAATAG	
MVK-S208A-F	GATAGACAACACCGTCgcTGCATACG	
MVK-S208A-R	CATGTTGCCGTATGCAgcGACGGTG	
MVK-T222A-F	GAGATAgCTCGGTTACAATCCAA	
MVK-T222A-R	TAACCGAGcTATCTCGCCTGAGC	
MVK-S263A-F	ATGAAGgCAGTGTTCAACGCCGT	
MVK-S263A-R	GAACACTGcCTTCATCGCATCAG	
MVK-T285A-F	GACGAGgCCTCAGTTACAGAAAA	
MVK-T285A-R	AACTGAGGcCTCGTCTTTAGACT	
MVK-S329A-F	CTTGTCgCCAAACTTACAGGAGC	
MVK-S329A-R	AAGTTTGGcGACAAGCTTGTGCT	
MVK-T332A-F	AAACTTgCAGGAGCTGGTGGCGG	
MVK-T332A-R	AGCTCCTGcAAGTTTGGAGACAA	
MVK-T342A-F	GTCCTCgCTCTATTACCAACCGG	
MVK-T342A-R	TAATAGAGcGAGGACGCAGCCGC	
MVK-253-F	attgGTCTCCTCTGAACATGAAGC	CRISPR/CAS9
MVK-253-R	aaacGCTTCATGTTCAGAGGAGAC	
MVK-927-F	attgGGTTAGCCACAGCTCAATCG	
MVK-927-R	aaacCGATTGAGCTGTGGCTAACC	
P2K1-QF	TGGAGTTTGTCAGGTCCATCG	Real-time qPCR
P2K1-QR	CTGAGGATCTTCTGCAGGCAA	
MVK-QF	TCCAAACTTACAGGAGCTGGTGG	
MVK-QR	AATGCCGTGAAACACTGAAAAC	
UBQ-QF	GGCCTTGTATAATCCCTGATGAATAAG	
UBQ-QR	AAGAAGAAGTTCGACTTGTCATTAGAA	
SAND-F	AACTCTATGCAGCATTTGATCCACT	
SAND-R	TGATTGCATATCTTTATCGCCATC	
WRKY40-QF	CCTCCCAAGAAACGCAAATC	
WRKY40-QR	AACCGCGCAGCTGAATG	
CPK28-QF	ACCCACGAGCACGGCTAA	
CPK28-QR	TTCTCTAACCCACGCATGTGAT	

Supplementary Table 2. Description of the processes and parameters applied to LC-MS chromatograms with MZmine ver.2.38 to obtain the metabolomic fingerprints of *Arabidopsis* root samples from both positive and negative ionization modes

		Parameters
**1**	**Baseline correction – RollingBall baseline corrector**	
	Chromatogram type	TIC
	Use m/z bins	No
	wm	25
	ws	25
**2**	**Mass detection** (exact Mass)	
	Noise level	1×10^3
**3**	**FTMS shoulder peak filter**	
	Mass Resolution	60,000
	Peak model function	Lorentzian
**4**	**Chromatogram builder**	
	Minimum time span	0.04
	Minimum height	1×10^3
	m/z tolerance	0.0005 m/z or 7ppm
**5**	**Smoothing**	
	Filter width	5
**6**	**Chromatogram deconvolution (local minimum search)**	
	Chromatographic threshold	40%
	Search minimum in RT range (min)	0.3
	Minimum relative height	30%
	Minimum absolute height	3000
	Minimum ratio of peak top/edge	1.5
	Peak duration range	0-1
**7**	**Normalization – Retention time normalizer**	
	m/z tolerance	0.0005 m/z or 7ppm
	RT tolerance	0.3
	Minimum Standard Intensity	1×10^5
**8**	**Chromatogram alignment (join alignment)**	
	m/z tolerance	0.0005 m/z or 6ppm
	Weight for m/z	80
	RT tolerance	0.25
	Weight for RT	40
**9**	**Gap filling (Peak Finder)**	
	Intensity tolerance	30%
	m/z tolerance	0.0005 m/z or 6ppm
	Retention time tolerance	0.25

	RT correction	Yes
**10**	**Metabolite Assignation**	
	m/z tolerance	0.001 m/z or 15ppm*
	RT tolerance	0.45*
**11**	**Data Exported**	Peak Area

RT, retention time; m/z, mass to charge ratio

* Metabolite assignation filtered a posteriori (Supplementary Data 3)
