# Supplementary material for: Activation of the plant mevalonate pathway by extracellular ATP
Source: Nat Commun. 2022 Jan 21;13:450. doi: 10.1038/s41467-022-28150-w (PMC8783019; doi:10.1038/s41467-022-28150-w)
Supplement: Supplementary file 2 — Description of Additional Supplementary Files [file 41467_2022_28150_MOESM2_ESM.pdf]

Description of Additional Supplementary Files

File name: Supplementary Data 1

Description: All peaks from mvk-1 volcano analysis of identified metabolites compared to ColQ (n=8 biological replicates, *$P < 0.05$, two-sided Student‟s t-test)

File name: Supplementary Data 2

Description: LC/MS dataset of ColQ and mvk-1

File name: Supplementary Data 3

Description: Measured and calibrated retention time (RT) and mass to charge ratio (m/z) of the deconvoluted ions in both negative and positive ionization modes assigned to metabolites with MZmine 2. RT and m/z of the measured standards included in the library are shown in the table. Error of m/z and RT of assigned ions to metabolites respect the m/z and RT of standards are shown before and after calibration. After the chromatogram builder and deconvolution algorithms of MZmine 2, several ions with the same exact mass may have been separated into two or more independent deconvoluted peaks presenting slightly different retention times and or m/z. The following table show all the peaks assigned to a molecular compound based on the exact mass of their parent ion (in negative or positive mode). For the final dataset of this study includes, all identified metabolic features assigned to a same metabolite were summed to finally have a single variable per metabolite. Last column of the table shows whether such metabolite match passed the matching filtering as described at the end of the table.
