# Supplementary material for: Activation of the plant mevalonate pathway by extracellular ATP
Source: Nat Commun. 2022 Jan 21;13:450. doi: 10.1038/s41467-022-28150-w (PMC8783019; doi:10.1038/s41467-022-28150-w)
Supplement: Supplementary file 6 — Reporting Summary [file 41467_2022_28150_MOESM6_ESM.pdf]

nature portfolio

Corresponding author(s): DBPR, NCOMMS-20-33356B

Last updated by author(s): 2021.12.17

Reporting Summary

Nature Portfolio wishes to improve the reproducibility of the work that we publish. This form provides structure for consistency and transparency in reporting. For further information on Nature Portfolio policies, see our Editorial Policies and the Editorial Policy Checklist.

Statistics

For all statistical analyses, confirm that the following items are present in the figure legend, table legend, main text, or Methods section.

n/a	Confirmed	
☐	☒	The exact sample size (*n*) for each experimental group/condition, given as a discrete number and unit of measurement
☐	☒	A statement on whether measurements were taken from distinct samples or whether the same sample was measured repeatedly
☐	☒	The statistical test(s) used AND whether they are one- or two-sided<br>*Only common tests should be described solely by name; describe more complex techniques in the Methods section.*
☐	☒	A description of all covariates tested
☐	☒	A description of any assumptions or corrections, such as tests of normality and adjustment for multiple comparisons
☐	☒	A full description of the statistical parameters including central tendency (e.g. means) or other basic estimates (e.g. regression coefficient) AND variation (e.g. standard deviation) or associated estimates of uncertainty (e.g. confidence intervals)
☒	☐	For null hypothesis testing, the test statistic (e.g. *F*, *t*, *r*) with confidence intervals, effect sizes, degrees of freedom and *P* value noted<br>*Give P values as exact values whenever suitable.*
☒	☐	For Bayesian analysis, information on the choice of priors and Markov chain Monte Carlo settings
☒	☐	For hierarchical and complex designs, identification of the appropriate level for tests and full reporting of outcomes
☒	☐	Estimates of effect sizes (e.g. Cohen's *d*, Pearson's *r*), indicating how they were calculated

Our web collection on statistics for biologists contains articles on many of the points above.

Software and code

Policy information about availability of computer code

Data collection	Arabidopsis genome version TAIR10 (for whole genome sequencing), Arabidopsis proteome data from NCBI database (for Mass spectrometry)
Data analysis	The data were analyzed using Trimmomatic version 0.32, Bowtie version 2, Samtools version 0.1.7, LinReg software version 11.0, Microsoft Excel 2020, Graphpad Prism 7, Image J, Thermo Scientific Proteome Discoverer v 2.2, MZmine 2 version 2.38, MetImp1.2, and MetaboAnalyst 4.0.

For manuscripts utilizing custom algorithms or software that are central to the research but not yet described in published literature, software must be made available to editors and reviewers. We strongly encourage code deposition in a community repository (e.g. GitHub). See the Nature Portfolio guidelines for submitting code & software for further information.

Data

Policy information about availability of data

All manuscripts must include a data availability statement. This statement should provide the following information, where applicable:

- Accession codes, unique identifiers, or web links for publicly available datasets
- A description of any restrictions on data availability
- For clinical datasets or third party data, please ensure that the statement adheres to our policy

All data supporting the findings of this study are included in this manuscript and its supplementary files or further materials can be obtain from the corresponding author upon request. The whole-genome sequencing, BiFC microscopy, and metabolites data are available via the following link (https://osf.io/5spkm/). Source data are provided with this paper.

Field-specific reporting

Please select the one below that is the best fit for your research. If you are not sure, read the appropriate sections before making your selection.

☒ Life sciences ☐ Behavioural & social sciences ☐ Ecological, evolutionary & environmental sciences

For a reference copy of the document with all sections, see nature.com/documents/nr-reporting-summary-flat.pdf

Life sciences study design

All studies must disclose on these points even when the disclosure is negative.

Sample size	Microsoft excel 2020 and Graphpad Prism 7 were used to estimate or calculate the sample size. The level of significance set at 5%. For ATP-induced calcium assay, 9-12 seedling plants in each group was finally analyzed.
Data exclusions	No data were excluded.
Replication	All experiments were reproduced to reliably support conclusions stated in the manuscript.
Randomization	Plants were randomly divided into experimental groups.
Blinding	Treatment of chemical compounds was carried out as both blinded and non-blinded experiments. In addition, at least three-person, independently tested the chemical compounds into wild-type and mutant plants.

Reporting for specific materials, systems and methods

We require information from authors about some types of materials, experimental systems and methods used in many studies. Here, indicate whether each material, system or method listed is relevant to your study. If you are not sure if a list item applies to your research, read the appropriate section before selecting a response.

Materials & experimental systems

n/a	Involved in the study	
☐	☒	Antibodies
☒	☐	Eukaryotic cell lines
☒	☐	Palaeontology and archaeology
☒	☐	Animals and other organisms
☒	☐	Human research participants
☒	☐	Clinical data
☒	☐	Dual use research of concern

Methods

n/a	Involved in the study	
☒	☐	ChIP-seq
☒	☐	Flow cytometry
☒	☐	MRI-based neuroimaging

Antibodies

Antibodies used	rabbit anti-phospho-p44/p42 MAPK antibody (Cell signaling technology, Cat.No.50-191-932), anti-HA-HRP (Sigma-Aldrich, Roche, Cat. No.12 013 819 001), anti-Myc-HRP (Santa Cruz, Cat.No.sc-40), Goat anti-rabbit-HRP (Sigma, Cat.No.12-348)
Validation	All antibodies were used in the system under study (assay and species) according to the profile of manufacturer.
